# *Operando* Fe dissolution in Fe–N–C electrocatalysts during acidic oxygen reduction: impact of local pH change†

**DOI:** 10.1039/d4ee01995d

**Published:** 2024-07-30

**Authors:** Angus Pedersen, Kavita Kumar, Yu-Ping Ku, Vincent Martin, Laetitia Dubau, Keyla Teixeira Santos, Jesús Barrio, Viktoriia A. Saveleva, Pieter Glatzel, Vinod K. Paidi, Xiaoyan Li, Andreas Hutzler, Maria-Magdalena Titirici, Antoine Bonnefont, Serhiy Cherevko, Ifan E. L. Stephens, Frédéric Maillard

**Affiliations:** a Imperial College London, Department of Materials, Royal School of Mines London SW7 2AZ UK i.stephens@imperial.ac.uk; b Imperial College London, Department of Chemical Engineering London SW7 2AZ UK; c Univ. Grenoble Alpes, Univ. Savoie-Mont-Blanc, CNRS, Grenoble-INP, LEPMI 38000 Grenoble France frederic.maillard@grenoble-inp.fr; d Forschungszentrum Jülich GmbH, Helmholtz-Institute Erlangen-Nürnberg for Renewable Energy (HI ERN) Cauerstraße 1 91058 Erlangen Germany; e Friedrich-Alexander-Universität Erlangen-Nürnberg, Department of Chemical and Biological Engineering Cauerstraße 1 91058 Erlangen Germany; f ESRF, The European Synchrotron 71 Avenue des Martyrs, CS40220 38043 Grenoble Cedex 9 France; g Laboratoire de Physique des Solides CNRS, Université Paris Sud 91405 Orsay France

## Abstract

Atomic Fe in N-doped C (Fe–N–C) catalysts provide the most promising non-precious metal O_2_ reduction activity at the cathodes of proton exchange membrane fuel cells. However, one of the biggest remaining challenges to address towards their implementation in fuel cells is their limited durability. Fe demetallation has been suggested as the primary initial degradation mechanism. However, the fate of Fe under different operating conditions varies. Here, we monitor *operando* Fe dissolution of a highly porous and >50% FeN_*x*_ electrochemical utilization Fe–N–C catalyst in 0.1 M HClO_4_, under O_2_ and Ar at different temperatures, in both flow cell and gas diffusion electrode (GDE) half-cell coupled to inductively coupled plasma mass spectrometry (ICP-MS). By combining these results with *pre*- and *post-mortem* analyses, we demonstrate that in the absence of oxygen, Fe cations diffuse away within the liquid phase. Conversely, at −15 mA cm^−2^_geo_ and more negative O_2_ reduction currents, the Fe cations reprecipitate as Fe-oxides. We support our conclusions with a microkinetic model, revealing that the local pH in the catalyst layer predominantly accounts for the observed trend. Even at a moderate O_2_ reduction current density of −15 mA cm^−2^_geo_ at 25 °C, a significant H^+^ consumption and therefore pH increase (pH = 8–9) within the bulk Fe–N–C layer facilitate precipitation of Fe cations. This work provides a unified view on the Fe dissolution degradation mechanism for a model Fe–N–C in both high-throughput flow cell and practical operating GDE conditions, underscoring the crucial role of local pH in regulating the stability of the active sites.

## Abstract 2

Fe cations produced during the reduction of O_2_ on Fe–N–C materials transform into Fe-oxides due to a local increase in pH.

## Graphical abstract

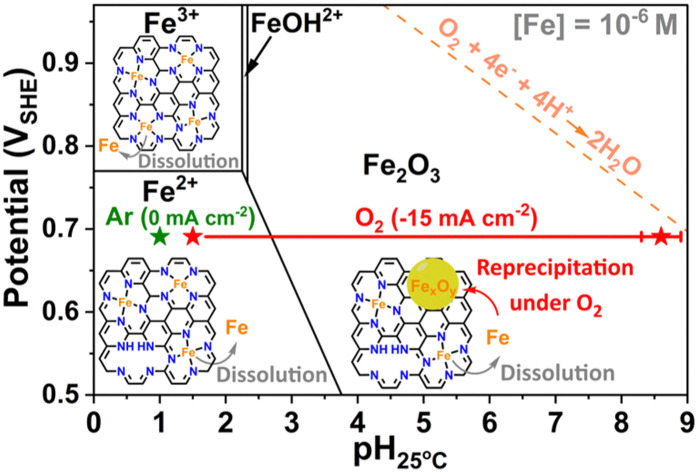

**Broader context**

Low-temperature proton exchange membrane fuel cells (PEMFC) fuelled by green hydrogen offer the potential for providing decarbonized energy. Platinum (Pt) is commonly used as a electrocatalyst, but due to its rarity and cost, minimizing its loading is crucial for widespread technology deployment. Atomically-dispersed iron atoms coordinated to nitrogen-doped carbon (Fe–N–C) are primary contenders to replace Pt as electrocatalysts for the O_2_ reduction reaction. While researchers have made huge inroads towards reaching parity between the two classes of catalysts in terms of activity, the durability of Fe–N–C falls far short of that of Pt. Our current study demonstrates that, at relevant current densities for PEMFC applications, iron atoms dissolve and then reprecipitate in the form of iron oxides. This outcome is a result of an increase in local pH, *i.e.* across the electrode | electrolyte interface, as indicated by results from online inductively coupled plasma mass spectrometry and a microkinetic model specifically developed for this study. Even at a moderate O_2_ reduction current density of −15 mA cm^−2^_geo_ at 20 °C, the local pH rises from 1 to 8–9. Given that practical electrochemical energy conversion and storage systems operate at significantly higher current densities, we expect substantial variations in pH in real fuel cells and water electrolyser devices. These pH changes will affect reaction kinetics, selectivity, and durability. Consequently, ensuring the alignment of local pH with bulk pH emerges as a crucial, albeit underexplored, factor for the sustainable operation of energy storage and conversion systems.

## Introduction

Low temperature proton exchange membrane fuel cells (PEMFCs) powered by green hydrogen provide a means to sustainable energy conversion for stationary and transport applications. Their widespread commercialization is partially limited by the cost of the platinum (Pt)-based nanoparticles supported on high surface area carbon (Pt/C) at the cathode, where oxygen reduction reaction (ORR) occurs. Single iron (Fe), cobalt (Co), manganese (Mn) or tin (Sn) atoms (and their combinations) coordinated to nitrogen-doped carbon (M–N–C, where M is the metal) exhibit the most promising non-precious metal activity for ORR.^1–5^ Of these, Fe–N–C has exhibited the greatest PEMFC performance.^6^ Still, ∼60–100 μmFe–N–C thick Fe–N–C cathodes are commonly used to compete with the PEMFC performance of ∼5 μm_Pt/C_ thick Pt-based cathodes,^7^ due mainly to Fe–N–Cs lower specific and volumetric active site density.^8–10^ Continuum modelling by Litster and coworkers found for high normalised site density Fe–N–C the cathode thickness should actually be <30 μm for max power density at 0.6–0.5 V and for max power.^11^ Employing these insights, Fe–N–C offer a potentially less expensive and less environmentally impactful alternative to Pt/C,^12,13^ although highly active Fe–N–C typically suffer from lower durability.^5,14^ Researchers have improved the stability of Fe–N–C by improved synthesis pathways, producing atomically dispersed active sites, rather than encapsulated nanoparticles, which induce instability.^15^ Most recently adding atomically thin protective coatings or reductive pyrolysis conditions has led to Fe–N–C durability beyond 300 h in PEMFC under H_2_/Air.^6,16^

However, Fe–N–C durability is still below commercial realization for transport applications (>5000 h)^17^ owing to several degradation routes,^18^ which can be separated into two categories. Firstly, support modification, such as oxidation of the N–C matrix,^2,19,20^ and N-protonation (especially for materials synthesized through pyrolysis under ammonia).^21^ Second is direct active metal atom modification by agglomeration/aggregation, and demetallation/dissolution.^19,22,23^ The demetallation of the active site can also take place indirectly through chemical or electrochemical corrosion of the N–C matrix.^24^ Steps can be taken to deconvolute these degradation pathways^25^ and also minimize them,^26,27^ or even temporarily reverse them by reactivation.^28^ However, studies point towards the demetallation of FeN_*x*_ active sites being the primary irreversible performance degradation mechanism in PEMFCs^6,29,30^ and the first step in the aggregation scenario.^22^

Inductively coupled plasma mass spectrometry (ICP-MS) is a highly sensitive technique which can provide time- and potential-resolved Fe dissolution profiles from Fe–N–C catalysts. Monitoring Fe dissolution from *ex situ* ICP-MS, in tandem with other characterization techniques, in rotating disc electrode (RDE)^31^ and PEMFC has revealed significant dissolution of Fe,^32^ although probing the mechanism requires *operando* measurements. In the first online flow cell ICP-MS study, Choi *et al.* suggested formation of insoluble ferric (Fe^3+^) species, which dissolve under PEMFC operating conditions (*E*_cathode_ < 0.7 V_RHE_) due to *operando* reduction to soluble ferrous (Fe^2+^) cations.^27^ This is in line with former *ex situ* ICP-MS findings of Zelenay and coworkers^31^ who suggested higher solubility of Fe^2+^ species in acid solutions compared to Fe^3+^ species. Previous online flow cell ICP-MS studies also provided critical information on the effects of pyrolysis atmosphere, bulk electrolyte pH, and catalyst modification on the extent of Fe dissolution.^19,27,33^

Nonetheless, flow cell ICP-MS studies are limited to low current densities, and cannot reproduce all the practical conditions occurring in an operating PEMFC device (O_2_ partial pressure and current density, lower relative humidity).^34^ In this respect, online gas diffusion electrode (GDE) ICP-MS is an adequate tool to simulate the environment of a PEMFC cathode more realistically, and gain PEMFC-relevant durability trends. For instance, Ehelebe *et al.* first demonstrated significantly lower dissolution of Pt/C catalysts in GDE configuration compared to flow cell systems due to varying mass transport conditions of Pt species,^35^ as previously proposed.^36^ Very recently, Choi and coworkers^26^ monitored *in situ* changes in active site density and *operando* Fe dissolution of a Fe–N–C under Ar and O_2_ at different temperatures using GDE ICP-MS cell in acidic conditions. From site density monitoring, the reduced turnover frequency confirmed a reactive oxygen species catalyzed carbon corrosion scenario.^24,37^ However, despite using a GDE, Choi and coworkers current densities at 0.6 V chronoamperometric holds (<10 mA cm^−2^_geo_) were comparable to values achievable in flow cell (∼1–2 mA cm^−2^_geo_), and not practical PEMFCs. They observed from *post-mortem* transmission electron microscopy (TEM) and energy dispersive X-ray spectroscopy (EDXS) elemental mapping that Fe deposited as Fe_*x*_O_*y*_ nanoparticles after O_2_ reduction in their Fe–N–C derived from microporous zeolitic imidazolate framework-8 (ZIF-8), confirming earlier findings from Kumar *et al.*^37^ Evidence of Fe_*x*_O_*y*_ nanoparticle formation in PEMFC-relevant conditions has previously been ascribed to highly active but unstable high-spin FeN_4_C_12_ moieties, *via* Mössbauer spectroscopy.^38^

Temperature is also a critical parameter for durability of Fe–N–C catalysts. Goellner *et al.* first evidenced that the rate of corrosion of a N–C matrix (150 square wave cycling between 0.9–1.4 V_RHE_, 3 s holds in RDE) increases 14-fold when temperatures increase from 20 to 80 °C. This resulted in 18-fold larger O_2_ reduction activity decay (at 0.8 V_RHE_), which was assigned to N–C corrosion.^39^ Carbon corrosion can be avoided at 25 °C in RDE by keeping potential <0.9 V_RHE_,^19^ although some carbon corrosion (<7 mA cm^−2^_geo_) is reported in PEMFC at 80 °C.^23^ Kumar *et al.* reported Fe cluster formation under load cycling (Ar-saturated 0.1 M H_2_SO_4_, 0.6–1.0 V_RHE_) at 80 °C, but did not observe Fe clusters at 60 °C, providing strong evidence of the effect of temperature on the fate of Fe species.^22^ Finally, we note that Osmieri *et al.* reported greater performance loss under air-fed *vs.* N_2_-fed PEMFC cathode (3 s holds at 0.95 and 0.6 V_vs anode_, 80 °C), although with no nanoparticle formation.^40^ Meanwhile Banham *et al.* qualitatively proposed a larger reaction zone in the Fe–N–C catalyst layer under air *vs.* O_2_ due to the difference in O_2_ concentration and also improved stability with lower equivalent ionomer due to improve ionic conductance.^41^ Therefore, conflicting characterisation results in literature could be due to operation conditions, (temperature, gas atmosphere, ionomer content and type, current densities, potential *etc.*), storage conditions,^42^ electrode preparation^43^ and synthesized Fe–N–C properties.^38^ Moreover, most of Fe–N–C catalysts studied by *operando* ICP-MS have consisted of low active site utilization Fe–N–C derived from ZIF-8. Our laboratory,^44^ and others,^45,46^ have highlighted that such catalysts display a predominantly or purely microporous structure. This limits the mass transport and electrochemical active site utilization (number of electrochemically accessible FeN_*x*_ sites to the total number of FeN_*x*_ sites) to typically <10%.^44–46^ This prompted us to revisit Fe dissolution and the fate of Fe in FeN_*x*_ active sites from our recently developed high FeN_*x*_ utilisation (>50%) Fe–N–C with high micro- and meso-porosity.^44^ This pore structure can facilitate mass transport of reactants for improved activity, while also enabling transport of dissolved Fe ions for *operando* ICP-MS detection.

The impact on Fe–N–C activity from changes in the (micro-) environment from RDE to GDE/PEMFC has been discussed.^47–49^ Local pH is one value which could vary between electrocatalysts and testing conditions, and is recognized to influence Fe–N–C activity.^50–52^ Meanwhile, the influence of pH on degradation is beginning to receive greater attention in modelling reaction mechanisms and dissolution trends.^53^ Local pH (at the interface between the working electrode and the bulk of the electrolyte) and its effects has been investigated and discussed quite extensively in electrochemical CO_2_ reduction;^54,55^ however, so far it has garnered limited experimental and theoretical evidence for ORR.^56–58^ This is because experimental pH probes (scanning probe, laser, RRDE, Raman, IR)^59^ have been limited to detecting proton concentrations away from the catalyst layer, are limited in pH ranges, cannot be easily transferred to high current devices such as GDEs and in some cases requires the addition of additives. Very recently Sauvé and coworkers proposed a new potential decay electrochemical technique for measuring pH within catalyst layers.^60^ Still, their method is restricted to providing an average interfacial pH of the electrode, relies on the H_2_/H^+^ equilibrium on Pt, and has an overestimation of the pH swing.

Meanwhile, kinetic modelling work by Zenyuk and Litster found during ORR increased pH along Pt mesopore channels, when devoid of Nafion and instead filled with water.^61^ It is worth considering that FeN_*x*_ active sites are proposed to be located within micropores,^23,45,62^ which are expected to be filled with water.^23^ Even so, Banham and coworkers’ experiments suggest that micropore flooding does not contribute significantly to PEMFC performance decay.^63^ Instead, kinetic models of Fe–N–C activity decay under different potentiostatic conditions in PEMFCs have been previously proposed,^23,64,65^ which has led to some debate.^66,67^ Still, to date these kinetic models of Fe–N–C have not factored in pH change and conditions in GDEs have not been considered.

In this work, we monitored Fe dissolution of a high electrochemical utilization Fe–N–C catalyst using *operando* flow cell and GDE ICP-MS. We found that the fate of Fe–N–C catalysts is determined by combined Fe demetallation, reactive oxygen species action (magnifying Fe demetallation) and local pH changes caused by ORR. We used a suite of complimentary *pre*- and *post-mortem* characterization techniques (SEM, TEM, STEM, EDXS, EELS, Raman spectroscopy, XRD, XPS, XANES) to illustrate changes in structure and chemistry; based on our experimental insights, we built a microkinetic modelling to interpret our observations.

## Experimental

### Fe–N–C Preparation

TAP 900@Fe and TAP 900@^57^Fe were prepared according to our previous work,^44^ with their synthesis also detailed in the ESI.†

### Online flow cell ICP-MS

The setup consisted of a homemade PEEK cell (Fig. S1, ESI†) with a three-electrode configuration using a glassy carbon rod as counter electrode (Sigradur grade G, HTW GmbH) and a leak-free Ag/AgCl/3.4 M Cl^−^ (ET072, eDAQ) as reference electrode. The Ag/AgCl/3.4 M Cl^−^ was calibrated *versus* reversible hydrogen electrode (RHE) *via* both a Hydroflex (Gaskatel) and a homemade Pt wire RHE. ^57^Fe in TAP 900@^57^Fe was used for online flow cell ICP-MS measurement to avoid interference from ArO^+^. The flow cell protocol and ICP-MS operation is detailed in the ESI† Fig. S2.

## GDE

### Electrode manufacture

The GDEs were prepared by doctor-blade coating an Fe–N–C ink onto a gas diffusion layer (GDL) including a microporous layer (Freudenberg, H23C8, 215.5 ± 6.5 μm). During the doctor-blade coating the temperature of the plate of automated film applicator (Zehntner, ZAA 2300) was at room temperature (23.5 ± 0.5 °C). The composition of the ink was 12 wt% solutes in a water (Milli-Q)/alcohol mixture, consisting of 68 wt% isopropanol (Supelco, EMSURE, ACS ISO), 17.6 wt% 1-Propanol, 13.6 wt% water (Milli-Q) and <0.8 wt% ethanol, where the latter three components are from the commercial Nafion solution (fuel cell store, D2021, 21 ± 1 wt% Nafion, 34 ± 2 wt% water, 44 ± 2 wt% 1-propanol, and < 2 wt% ethanol). The solute fraction comprised 41.3 wt% of TAP 900@Fe material and 58.7 wt% of Nafion. Due to the high mesopore volume of TAP 900@Fe,^44^ a relatively high ionomer to Fe–N–C weight ratio of 1.42 : 1 was used to ensure utilization of the catalyst layer. Optimisation of the ionomer:catalyst ratio has been considered in PEMFC in a separate study^68^ and its impact on Fe dissolution will be investigated in a future work. After 30 min of stirring and 1 h of sonication (100 W VWR Ultrasonic Cleaner USC 500 THD) at *T* < 30 °C, the ink was constantly stirring until deposition. After the ink deposition onto the GDL, the samples were dried at room temperature (21 ± 2 °C) under atmospheric pressure until testing. The catalyst layer loading was 0.86 ± 0.15 mg_FeNC_ cm^−2^_geo_, as determined by weighing the GDE before and after Fe–N–C coating. The catalyst layer thickness was 58 ± 4 μm, as measured by a micrometer (Helios Preisser, 0912501).

### Online GDE ICP-MS

Prior to electrochemical testing, GDEs were immersed in ultrapure water for 1 hour. The electrolyte, reference and counter electrodes were 0.1 M HClO_4_ (Suprapur, Sigma Aldrich), Ag/AgCl (inner and outer compartments filled with 3 M KCl and 0.1 M HClO_4_, respectively, Metrohm) and Ti/Ir mixed oxide grid (METAKEM), respectively. Ag/AgCl/3 M KCl was calibrated every day at the temperature of interest (*E*_Ag/AgCl/Cl^−^_ = 0.316 ± 0.011 V_RHE_ at 20 °C and *E*_Ag/AgCl_ = 0.297 ± 0.013 V_RHE_ at 75 °C). A gas humidification system built with two gas washing bottles (Duran) and a heating plate (IKA^TM^ RCT basic hot plate stirrer) was used to heat the purged gases to 75 °C. The GDE half-cell was heated to 74 ± 1 °C using an electrolyte recirculation system *via* a heating bath (AQUAline, LAUDA). In GDE, following the previously reported protocol,^69^ 100% post *iR* correction was applied for O_2_ measurements, while for Ar measurements, 50% was applied *in situ* and 50% post Ar experiment. Details of GDE ICP-MS operation and protocol are detailed in the ESI,† Table S1 and Fig. S3. The online Fe dissolution was measured with our previously reported GDE ICP-MS setup,^35,70^ shown in Fig. S4 (ESI†).

### Microkinetic modelling

A one-dimensional model was developed to describe pH distribution in the catalyst layer. This model encompasses a system of partial differential equations (ESI†) that account for the transport of Fe and protons in the electrolyte and the 60 μm thick catalyst layer, as well as the proton consumption by the ORR and the dissolution/precipitation of Fe cations in the catalyst layer. The modelling is based on the following assumptions:

(i) The ORR kinetics in the GDE is limited by proton mass transport, with the O_2_ concentration being uniform in the catalyst layer.

(ii) The dissolution and precipitation of Fe cations occurs in the water present in the pores *i.e.*, the precipitation of Fe cations is not influenced by the Nafion ionomer in the catalyst layer:Fe^3+^ + 3H_2_O → Fe(OH)_3_ + 3H^+^

(iii) Due to the pronounced difference in complexation constants, only Fe^3+^ cations are expected to precipitate.^71^ If Fe^2+^ cations are dissolved in water, they will anyway thermodynamically be oxidized into Fe^3+^ cations by O_2_.^72^

(iv) Based on the GDE ICP-MS data at 20 °C that will be discussed later, the rate of production of dissolved Fe ions is assumed to be approximately two times faster in O_2_ than in Ar GDE experiments.

(v) A homogeneous potential distribution is assumed in the catalyst layer.

## Results

### Comparing TAP 900@Fe and TAP 900@^57^Fe RDE ORR activity

Thorough *ex situ* characterization of TAP-derived materials was carried out in our previous work.^44^ However, some comparisons between TAP 900@^57^Fe and TAP 900@Fe were missing. Considering O_2_ reduction, reduced activity has previously been reported for ^57^Fe enriched Fe–N–C samples compared to Fe–N–C prepared in the same manner but with natural abundance Fe precursor.^73^ The RDE O_2_ reduction mass activity for TAP 900@^57^Fe and TAP 900@Fe can be found in Fig. S5a and b (ESI†). The kinetic region and mass activity at 0.8 V_RHE,iR-free_ in O_2_-saturated RDE is lower in TAP 900@^57^Fe compared to previously reported TAP 900@Fe,^44^ with 3.77 ± 0.54 and 5.01 ± 0.79 A g_FeNC_^−1^, respectively (Fig. S5a, ESI†). The lower activity with ^57^Fe enrichment follows the previous report.^73^

### Online flow cell ICP-MS

**Fig. 1:**
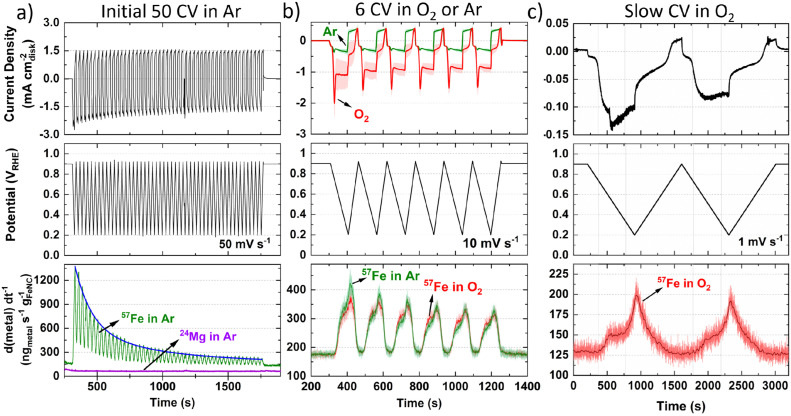
Online flow cell ICP-MS metal dissolution during (a) Initial 50 CVs at 50 mV s^−1^ under Ar-saturated 0.1 M HClO_4_ with 0.2 mg_FeNC_ cm^−2^. Blue line represents fitted exponential decay. (b) Six CV at 10 mV s^−1^ under Ar-(green) and O_2_-(red) saturated conditions. Error represents standard deviation from four separate measurements. (c) Two slow CV scans at 1 mV s^−1^ under O_2_-saturated 0.1 M HClO_4_ with 0.2 mg_FeNC_ cm^−2^. Dark red line represents fast Fourier transform smoothed data.

Moving to *operando* flow cell ICP-MS measurements in 0.1 M HClO_4_, TAP 900@^57^Fe was used to avoid polyatomic interference from ArO^+^ and maximize spectrometric signal. The setup and experimental protocol are depicted in Fig. S1 and S2 (ESI†), respectively. First, ICP-MS calibration, electrochemical impedance spectroscopy and open circuit potential (OCP) measurements were conducted to ensure correct installation and operation. Next, 50 fast (50 mV s^−1^) cyclic voltammograms (CVs) between 0.925–0.200 V_RHE_ were measured in Ar-saturated electrolyte to allow the catalyst to reach a stable electrochemical and dissolution measurement (Fig. 1a, 0.2 mg_FeNC_ cm^−2^_geo_). Mg was also monitored during the initial 50 cycles due to its use as a templating agent during synthesis, with 0.06 wt% detected from *ex situ* ICP-MS in our previous work.^44^ Mg dissolution did not vary with potential (Fig. 1a) and so is not considered further. Meanwhile, the rate of Fe dissolution followed an exponential decay.

Considering the effect of increased Fe–N–C loading, the amount of ^57^Fe dissolution follows a linear trend over the initial 50 CVs (Fig. S6a, ESI†). The percentage of total ^57^Fe detected increases from 7.5 ± 2.9% to 15.2 ± 3.3% as catalyst loading increases from 0.05 to 0.40 mgFe–N–C cm^−2^_geo_, with 11.3 ± 5.6% at 0.20 mgFe–N–C cm^−2^_geo_ (Fig. S6b, ESI†). This finding appears counterintuitive as one would expect either an equivalent percentage of Fe detected relative to the loading, or even a reduced percentage of detected Fe, due to reduced active site utilization with increasing thickness of the catalyst layer. It is also worth noting that there is a constant 130 ng_Fe_ g_FeNC_^−1^ s^−1 57^Fe concentration observed when held at 0.9 V_RHE_ (Fig. 1a–c), which was also the OCP of the TAP 900@^57^Fe catalyst.

After the initial 50 CVs at 50 mV s^−1^, six CVs were conducted at 10 mV s^−1^ under Ar and then O_2_-saturation. With increasing TAP 900@^57^Fe loading under O_2_-saturation, the limiting current density (below 0.65 V_RHE_) only incrementally increases. This slight increase can be explained by the increasing thickness of the catalyst layer with loading, which penetrates deeper into the flowing O_2_-saturated electrolyte. Meanwhile, between 0.65–0.80 V_RHE_ there is an increasing O_2_ reduction peak in the cathodic direction (Fig. S7a, ESI†). This is caused by a build-up of O_2_ concentration locally in the catalyst layer while scanning the potential region of 0.800–0.925 V_RHE_, where very little ORR is observed.

Under Ar-saturated conditions the current density increases proportional to the catalyst loading; we note there is an increasing peak on the cathodic scan (Fig. S7b, ESI†). We attribute this cathodic peak to the reduction of trace O_2_, arising from air ingress at the junction of the Kalrez O-ring and cell (or cavitation from the peristaltic pump). Still, the amount of O_2_ appears negligible. Normalizing the ^57^Fe detected to charge passed and catalyst loading shows the amount of ^57^Fe detected is constant under O_2_ but increases with reduced catalyst loading under Ar (Fig. S7c and d, ESI†). Meanwhile, the amount of ^57^Fe detected is equivalent under either gas saturation, with 1.3–2.0% of total ^57^Fe detected, and linear dependence with Fe–N–C loading (Fig. S7e and f, ESI†). Focusing on the dissolution at 0.2 mg_FeNC_ cm^−2^_geo_, similar profiles are observed under Ar and O_2_-saturation (Fig. 1b).

To better distinguish the Fe dissolution features, slow CVs (1 mV s^−1^) were conducted under O_2_-saturation (Fig. 1c). The slow scans show two onsets of ^57^Fe dissolution above background levels on the cathodic scan at *ca.* 0.72 and 0.33 V_RHE_ (Fig. 1c).

**Fig. 2:**
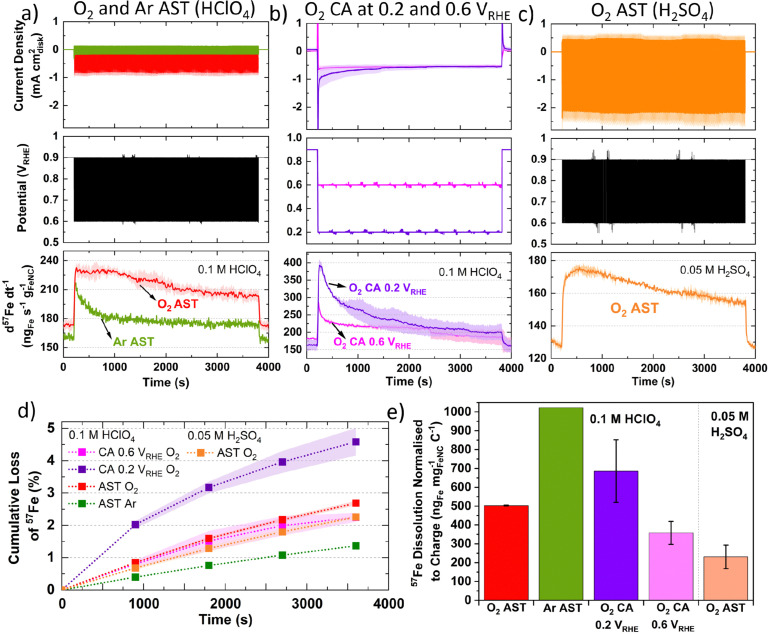
Online flow cell ICP-MS of TAP 900@^57^Fe (a) AST (3 s hold at 0.9 and 0.6 V_RHE_) under Ar- and O_2_-saturated 0.1 M HClO_4_. (b) 0.2 and 0.6 V_RHE_ CA in O_2_-saturated 0.1 M HClO_4_ (c) AST under O_2_-saturated 0.05 M H_2_SO_4_. Lighter shaded region represent error from two measurements. (d) Cumulative Fe loss. (e) Charge normalized Fe dissolution over varying stability test conditions. All tests over 1 h with 0.2 mg_FeNC_ cm^−2^_geo_. Error in figures represent two repeat measurements.

To evaluate differences in Fe detection and profiles over a longer period, 1 h AST or chronoamperometry (CA) were recorded in 0.1 M HClO_4_ (Fig. 2a and b) or 0.05 M H_2_SO_4_ (Fig. 2c). Greater ^57^Fe loading-normalized concentration is observed over the course of the AST under O_2_ than Ar. ^57^Fe concentration follows a slow decline under O_2_ and rapid plateau above baseline under Ar (Fig. 2a). 2.7 ± 0.1% of total ^57^Fe is detected during O_2_ AST (Fig. 2d), with a charge normalized Fe dissolution of 503 ± 3 ng_Fe_ mg_FeNC_^−1^ C^−1^ (Fig. 2e). Meanwhile, half ^57^Fe concentration is observed under Ar AST (Fig. 2d and Fig. S8, ESI†); however, normalizing to the total charge passed shows approximately double, with 1022 ng_Fe_ mg_FeNC_^−1^ C^−1^ (Fig. 2e). *Pre*- and *post-mortem* bright-field TEM of these samples shows no formation of detectable nanoparticles under Ar or O_2_ (Fig. S9, ESI†), indicating all Fe demetallation leads to dissolution at 25 °C, in agreement with former findings of Kumar *et al.*^37^

CA under O_2_ at 0.2 V_RHE_ shows a large initial spike in ^57^Fe concentration, which then decays over time, while CA at 0.6 V_RHE_ shows a smaller spike and lower overall dissolution (Fig. 2b). The initial spike in ^57^Fe concentration may be related to double layer charging and rapid change in potential. After 30 min, the current density and Fe dissolution are equivalent at 0.2 and 0.6 V_RHE_ CA. CA at 0.2 V_RHE_ ends with 4.6 ± 0.4% of total ^57^Fe and 686 ± 166 ng_Fe_ mg_FeNC_^−1^ C^−1^. This is approximately double the values at 0.6 V_RHE_, with 2.2 ± 0.1% ^57^Fe and 358 ± 61 ng_Fe_ mg_FeNC_^−1^ C^−1^ (Fig. 2e). This correlates with the observations from Fig. 1c, where greatest Fe dissolution occurs around 0.20 V_RHE_.

In 0.05 M H_2_SO_4_ instead of 0.1 M HClO_4_, O_2_ AST shows a similar dissolution profile, with lower Fe detection but higher O_2_ current densities (Fig. 2c). This difference in current is unexpected as O_2_ solubility is comparable at these acid concentrations. Meanwhile the total ^57^Fe loss is 2.3 ± 0.1% in 0.05 M H_2_SO_4_ and slightly higher in 0.1 M HClO_4_ with 2.7 ± 0.1% (Fig. 2d). However, the charge normalized Fe dissolution is less than half in 0.05 M H_2_SO_4_, at 231 ± 63 ng_Fe_ mg_FeNC_^−1^ C^−1^ (Fig. 2e). The presence of sulfate *versus* perchlorate will affect the Fe speciation and the mobility of Fe species.^74^ Additionally, the second acidity of H_2_SO_4_ is weak (*K*a_1_ = 10^+3^, *K*a_2_ = 1.26 × 10^−2^ = [H^+^][SO_4_^2−^]/[HSO_4_^−^]) compared to HClO_4_, which completely dissociates (*K*a = 10^+9^ = [H^+^][ClO_4_^−^]/[HClO_4_]).^75^ These results warrant future studies on Fe dissolution rates in HClO_4_ and H_2_SO_4_ with varying pH.

### GDE O_2_ reduction and degradation

While flow cell measurements proved insightful, the degradation rate in real PEMFCs may be different due to higher ORR rates and therefore ORR charge passed. To reach higher current densities and conditions comparable to PEMFCs, TAP 900@Fe was tested in a GDE half-cell coupled to online ICP-MS in 0.1 M HClO_4_ at 21 ± 1 °C and 74 ± 1 °C, denoted as 20 and 75 °C herein.

**Fig. 3:**
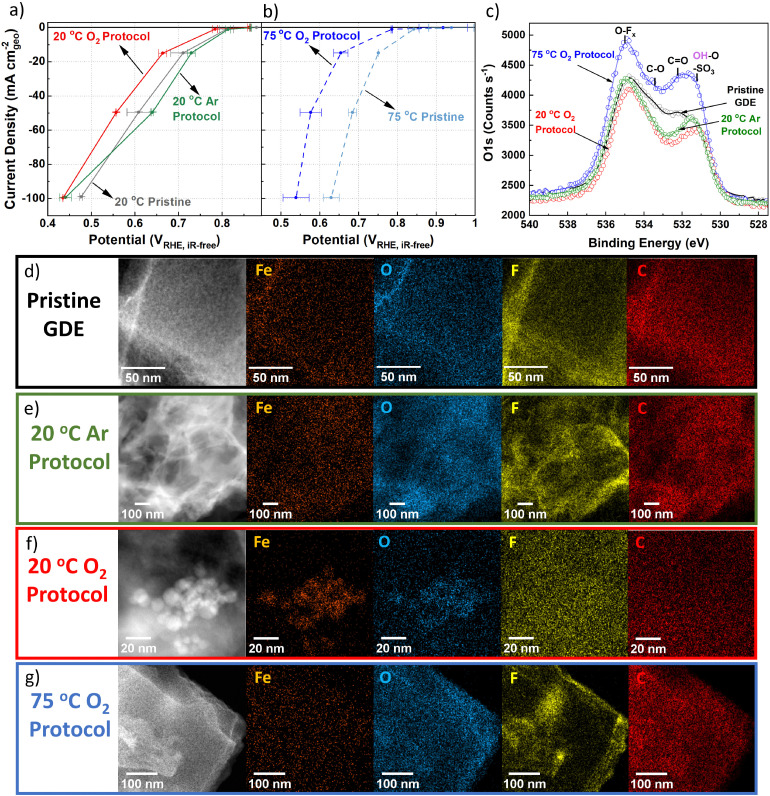
GDE polarization curves in 0.1 M HClO_4_ with 0.7–1.0 mg_FeNC_ cm^−2^_geo_ for (a) 25 °C O_2_ reduction in GDE for pristine TAP 900@Fe, and after O_2_ and Ar AST. (b) 75 °C O_2_ reduction in GDE for pristine TAP 900@Fe and after 75 °C O_2_ AST. Error represents two repeat measurements. 100% post iR correction was applied for O_2_ measurements. Comparison of pristine and post protocol GDEs (c) O 1s XPS (d–g) HAADF-STEM and STEM-EDXS.

Based on flow cell results, 50 CVs under Ar-saturation (50 mV s^−1^, 0.9–0.2 V_RHE_, Fig. S10, ESI†) were initially carried out to remove loosely bound Fe. O_2_ reduction was measured in GDE half-cell before and after AST tests (Fig. 3a and b). Catalyst loadings varied between 0.7 to 1.0 mg_FeNC_ cm^−2^_geo_, consequently corresponding mass activity plots are shown in Fig. S11a and b (ESI†). Compared to initial 20 °C O_2_ reduction, after 20 °C Ar AST there is an apparent improvement in O_2_ reduction performance at current densities up to −50 mA cm^−2^_geo_ (Fig. 3a). This is assigned to improved wetting of TAP 900@Fe during the 20 °C Ar AST. Meanwhile, 20 °C O_2_ AST led to noticeable performance degradation after only 200 cycles, with potential shift at −50 mA cm^−2^_geo_ of −50 ± 30 mV (from 0.61 ± 0.03 to 0.56 ± 0.00 V_RHE,iR-free_) compared to pristine 20 °C TAP 900@Fe (Fig. 3a). At −50 mA cm^−2^_geo_, 75 °C GDE pristine TAP 900@Fe shows an improved O_2_ reduction potential of 0.68 ± 0.01 V_RHE,iR-free_. Meanwhile, 75 °C O_2_ 200 AST cycles results in severe degradation to 0.58 ± 0.03 V_RHE,iR-free_ (Fig. 3b).

### Pre- and post GDE protocol characterisation

XPS peak fitting of fresh GDE C 1s and O 1s spectra is provided in Fig. S12a and b (ESI†) with comparison of O 1s between pre and post GDE protocol in Fig. 3c. Comparison of O 1s XPS spectra for fresh GDE and after 20 °C Ar and O_2_ protocols shows comparable total O 1s of 8.9–8.1 at% (Fig. S12c and Table S3, ESI†), with slight reductions in C=O and C–O peaks for AST samples (Fig. 3c). We tentatively assign this to the removal of carbon surface oxides during the initial 50 CVs. Meanwhile, after 75 °C O_2_ protocol, a clear overall O 1s increase is found, equivalent to 12.2 at% O 1s (Fig. S12c, ESI†). There is less discernible change in the C 1s spectra, aside from reduction in C–N and C–C and increase in CF2 in all AST samples compared to the pristine TAP 900@Fe GDE (Fig. S13, ESI†). Raman spectra (Fig. S14, ESI†) for pristine and post Ar and O_2_ 20 °C GDE protocols show no discernable difference (*I*_d_/*I*_g_ = 1.02–1.03, based on peak height), while there is a slight increase after 75 °C O_2_ protocol (*I*_d_/*I*_g_ = 1.05), indicating a minor increase in defects density in the carbon structure.

Within the pristine TAP 900@Fe GDE no visible nanoparticles >2 nm are detected using HAADF-STEM and STEM-EDXS spectrum imaging (Fig. 3d and Fig. S15a, ESI†); however, sub-nanometric Fe clusters below the detection limit of the microscope could be present, as observed in separate higher resolution HAADF-STEM measurements (Fig. S16, ESI†). After 20 °C Ar protocol, one large Fe_*x*_O_*y*_ nanoparticle is detected in the spectrum image, while, at higher magnification, small clusters are observed (Fig. 3e and Fig. S15b, ESI†). Numerous Fe nanoparticles are observed following 20 °C O_2_ protocol in GDE, which are assigned to Fe_*x*_O_*y*_ based on overlaying the Fe and O EDXS mapping (Fig. 3f and Fig. S15c, ESI†). HAADF-STEM combined with EDXS and EELS reveals clusters containing Ca and Fe in fresh and post Ar and O_2_ AST GDE (Fig. S16, ESI†). The presence of Ca remains unexplained, as we consistently used MilliQ water for all our electrochemical experiments and rinsing steps. No trace of Ca was also detected in the native catalyst. We therefore attribute it to contamination by tap water. The peak at 695 eV is from Fe–K. STEM-EELS analysis in regions without Fe particles cannot resolve any Fe peak (Fig. S16, ESI†), likely owing to the concentration of FeN_*x*_ sites being below the limit of detection.

Post 75 °C O_2_ protocol no large Fe_*x*_O_*y*_ particles are seen from EDXS and limited Fe clusters from HAADF-STEM (Fig. 3g and Fig. S15d, ESI†). No significant change from the pristine TAP 900@Fe structure is observed after 20 °C O_2_ and Ar protocols, (Fig. S17a–c, ESI†); however, after 75 °C O_2_ protocol a denser particle structure is observed (Fig. S17d, ESI†).

XRD on *post-mortem* GDE AST samples was conducted to try and deduce the type of Fe_*x*_O_*y*_, however either the lack of crystallinity, small particle size and/or low concentration meant no sharp peaks relating to Fe particles could be identified (Fig. S18, ESI†). The peak at 18.0° is assigned to polytetrafluoroethylene, which arises from the Nafion backbone. It is worth mentioning that pristine TAP 900@Fe does not show a graphite peak at ∼25.6° (002), suggesting its amorphous or graphene-like structure, with an average of single atomic layers found from previous Raman analysis.^76^

Normalized absorption and first derivative XANES of fresh TAP 900@Fe powder and GDE ink, plus post Ar and O_2_ 25 °C protocols, are compared to references of Fe foil, FeO and Fe_2_O_3_ in Fig. S19a and d (ESI†). A positive shift of center of mass of the pre-edge in TAP 900@Fe ink and after Ar and O_2_ protocols signifies an increase of oxidation state, while their decrease in intensity is related to a change in local coordination of Fe. TAP 900@Fe GDE ink displays a near identical spectra to post 25 °C O_2_. This suggests changes in Fe coordination and oxidation state between TAP 900@Fe powder and its ink Post Ar protocol shows a lower rising edge position indicating a lower average Fe oxidation state, or change in bond length and/or coordination change.

### Online GDE ICP-MS

**Fig. 4:**
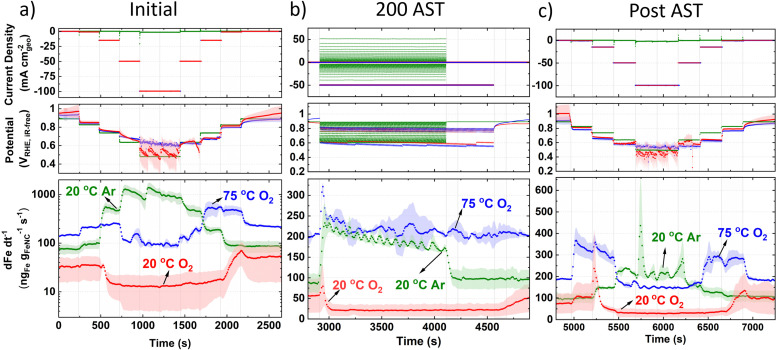
Fe concentration under Ar and O_2_ at 20 °C and O_2_ at 75 °C in online GDE ICP-MS with 0.1 M HClO_4_ and 0.7–1.0 mg_FeNC_ cm^−2^_geo_ (a) prior to AST (note *y*-axis is log-scale only in (a)). (b) 200 AST cycles. Under O_2_, the current was held for 3 s intervals 200 times at −0.05 and −50 mA cm^−2^_geo_, corresponding to *ca.* 0.85 and 0.6 V_RHE,iR-free_, respectively. Under Ar, the potential was held for 3 s intervals 200 times between 0.9 and 0.6 V_RHE,iR-free_. (c) Post AST. Error represents two repeat measurements.

To elucidate the Fe dissolution mechanisms in a practical device, online GDE ICP-MS was measured before, during and after the AST (Fig. 4) for each of the conditions. It is observed that the baseline Fe concentration is high even after the preliminary 50 CVs in Ar (50 mV s^−1^).

For initial 20 °C Ar (Fig. 4a), Fe concentration above baseline occurs at 0.83 V_RHE,iR-free_ and reaches a maximum concentration between 0.64–0.48 V_RHE,iR-free_. Returning to 0.83 V_RHE,iR-free_, Fe concentration returns to baseline levels. Initial 20 °C O_2_ current step holds (Fig. 4a) show a lower baseline Fe concentration than 20 °C Ar. A fall in Fe concentration below baseline levels is observed when increasing current density from −1 to −15 mA cm^−2^_geo_, corresponding to 0.85 ± 0.02 to 0.80 ± 0.01 V_RHE,iR-free_, respectively. When returning anodically to hold at −1 mA cm^−2^_geo_, Fe concentration increases and only begins falling back to baseline once returning to hold at −0.05 mA cm^−2^_geo_. Initial 75 °C O_2_ current hold measurements show a higher baseline Fe concentration, with increased Fe concentration during holds at −1 to −15 mA cm^−2^_geo_. Fe concentration then returns to approximate baseline values during holds at −50 and −100 mA cm^−2^_geo_, corresponding to 0.66 ± 0.04 and 0.62 ± 0.04 V_RHE,iR-free_, respectively. Returning anodically to holds at −15 and −0.6 mA cm^−2^_geo_ results in increased Fe concentration.

Moving to online AST monitoring (Fig. 4b), 20 °C Ar shows increased Fe concentration at the beginning of the AST. Fe concentration then gradually decreases over time and falls back to baseline levels after the AST. To note, AST O_2_ have the same number of cycles (200) in protocol as Ar (3 s holds at each potential), but O_2_ ASTs took a longer duration because of the additional time to switch the applied current ranges between 3 s holds, which is not required in Ar AST protocol. During the AST, 20 °C O_2_ shows a similar Fe concentration profile to current hold prior to AST (Fig. 4a), with an initial Fe concentration spike, followed by reduced Fe concentration below baseline levels. Fe concentration then returns to baseline levels post AST, without displaying a dissolution spike. 75 °C O_2_ also shows an initial spike in Fe concentration at the beginning of the AST, but then maintains baseline Fe concentration values during and post AST with no discernable change.

Post AST (Fig. 4c), 20 °C Ar show Fe concentration significantly decreases across the whole potential range compared to prior to the AST (Fig. 4a). This suggests unstable Fe species have been depleted over the AST. Current step holds after 20 °C O_2_ AST show a similar Fe concentration profile to measurements prior to AST, although a higher Fe concentration spike is observed post AST when stepping from −1 to −15 mA cm^−2^_geo_ (Fig. 4c). Post AST 75 °C O_2_ shows a symmetric Fe concentration profile when increasing and decreasing current. 75 °C O_2_ GDE ICP-MS results correlate with HAADF-STEM and STEM-EDXS observations (Fig. S15d, ESI†), where more Fe has dissolved rather than redeposited as particles, as is the case from 20 °C O_2_.

## Discussion

We now discuss all the results with the aim of establishing similarities or differences between the trends observed on our catalytic material and others.

### Flow Cell ICP-MS

Our experiments in flow cell ICP-MS first confirm that the dissolution of Fe atoms is indeed the predominant degradation mechanism in this type of catalyst. In CV, two well-defined Fe dissolution peaks can be observed, with onset of 0.73 and 0.33 V_RHE_ on the cathodic scan (Fig. 1c). The two ^57^Fe concentration peaks could represent two different Fe species dissolving at different potentials, or different dissolution process with different formal potentials. Only one Fe concentration peak was resolved by Santori *et al.*, with an onset of Fe concentration at *ca.* 0.75 V_RHE_ for their Ar-pyrolysed Fe–N–C in O_2_-saturated 0.1 M H_2_SO_4_ at 2 mV s^−1^ (data reproduced in Fig. S20, ESI†).^33^ Meanwhile Choi *et al.* observed the onset of increased Fe concentration at 0.77 V_RHE_, with two distinguishable Fe dissolution peaks,^19^ as observed here. The potential at which peak Fe concentration occurs is not discussed as this depends on mass transport, which changes with the electrochemical cell design and operating conditions.

We note that the onset of increased ^57^Fe concentration at *ca.* 0.73 V_RHE_ on the cathodic scan (Fig. 1c) coincides with the onset of the quinone–hydroquinone redox on the cathodic scan post 8000 O_2_ AST at 80 °C (Fig. 2c), and the second peak onset of increased ^57^Fe concentration at 0.33 V_RHE_ on the cathodic scan coincides with the onset of the second reversible redox on the cathodic scan. Our observations suggest that the stability of the Fe centre may be intrinsically linked to the chemistry of the surrounding ligands; this notion is analogous to relationships observed by others between the catalytic activity and the chemistry of the surrounding ligands.^24,77^

We also note an initial exponential decay in Fe concentration (Fig. 1a), which was also observed by Choi *et al.* for their Fe–N–C catalyst.^19,27^ In our case maximum Fe concentration is observed instantaneously upon potential cycling in Ar, whereas in the report of Choi *et al.* maximum Fe concentration is reached after 2–3 CVs.^19,27^ This could be due to the vastly different catalyst structures between our highly micro- and mesoporous TAP 900@^57^Fe with high active site utilization,^44^ and the bulky particle and predominantly microporous ZIF-8 derived Fe–N–C of Choi *et al.*^19,27^ Alternatively, it could arise from mass transport effects from slow residence time in Choi *et al.*'s flow cell design. The structure of unmodified microporous ZIF-8 derived materials would have impeded mass transport, low active site utilization and therefore delayed detection of Fe dissolution. Differences in experimental setup and residence time calibration in this work and that of Choi *et al.* could also contribute to the observed time difference in Fe concentration detection.

Choi *et al.*^27^ detected ∼3% of total Fe over their initial 20 CVs in Ar-saturation (100 mV s^−1^, 0.8 mgFe–N–C cm^−2^) for their purely microporous ZIF-derived wet synthesis Fe–N–C containing Fe_*x*_C and Fe particles. Meanwhile their dry synthesis or post chemical or electrochemical modification significantly reduced the initial Fe dissolution.^27^ On the other hand, after 50 CVs (50 mV s^−1^, 0.4 mgFe–N–C cm^−2^), TAP 900@^57^Fe shows 15.2 ± 3.3% Fe detected. This again points to the different porosity and structure in TAP- and ZIF-derived materials, leading to different accessibility of Fe sites. It also appears that while our decoupled wet-synthesis approach for TAP 900@Fe avoided the formation of Fe particles,^44^ it still leads to significant Fe dissolution during initial CVs. Additionally, it should be noted, according to our previous *ex situ* TAP900@^57^Fe Mössbauer assignments, *ca.* 11% of the Fe existed as inactive FeCl_2_·4H_2_O.^44^ This species may represent some or all of the initially dissolved Fe species.

In Fig. 2a, the rapid decay and plateau in Ar-saturation may be explained by the sudden step in potential, causing dissolution of inactive Fe species, with varying residence time of Fe across the catalyst layer. Meanwhile in O_2_-saturation the gradual decrease in Fe concentration is assigned from the decreasing concentration of highly active but unstable Fe species which dissolve during the O_2_ reduction cycle. Such unstable and dissolving FeN_*x*_ sites have been previously assigned to high spin Fe^3+^N_*x*_ sites from ^57^Fe Mössbauer spectroscopy,^38^ which were previously found to make up the most significant portion of Fe species in TAP 900@^57^Fe.^44^

Results from Fig. 1b (Fig. S7e and f, ESI†) suggest that the Fe concentration is independent of O_2_ reduction under cyclic voltammetry (0.9–0.2 V_RHE_ at 10 mV s^−1^) in flow cell. This is contrary to what is observed in Fig. 2a, where detected Fe concentration is greater under O_2_ than Ar under AST (step from 0.9 to 0.6 V_RHE_ with 3 s potential holds) flow cell conditions. These different Fe concentrations may be due to either the different potentials scanned (AST: 0.9–0.6 V_RHE_*versus* CV: 0.925–0.2 V_RHE_), the potential scanning protocol (AST: 3 s square wave voltammetry holds *versus* CV: 10 mV s^−1^), or 6 CVs not providing enough cycles to distinguish changes in Fe concentration. Unfortunately, the ICP-MS drift during flow cell operation prevented running a greater number of CVs to test this hypothesis.

Considering Fig. 2e and Fig. S8 (ESI†), Zelenay and coworkers also observed from *ex situ* ICP-MS that HClO_4_ dissolved more Fe from their polyaniline-derived Fe–N–C than H_2_SO_4_, which they attributed to differences in solubility of Fe perchlorates and sulfates.^31^ We suggest this observation could also be attributed to the stronger SO_4_^2−^ binding on the Fe site,^78^ whereas ClO_4_^−^ has been proposed to mimic non-specifically adsorbing properties of perfluoro sulfonic acid ionomers.^79^ If true, this would imply AST measurements in H_2_SO_4_ in RDE and GDE would lead to slower Fe–N–C degradation than in HClO_4_ (at the same pH), when Fe dissolution is the main degradation mechanism.

The different current density under 0.1 M HClO_4_ and 0.05 M H_2_SO_4_ (Fig. 2a and c) may be related to kinetic effects of the proton donor.^80^ Additionally, at 0.8 V_RHE_ Fe–N–Cs have recently been reported to possess 1.3–2.9 higher mass activity in H_2_SO_4_ than HClO_4_.^78^

### GDE ICP-MS

Under initial Ar in GDE ICP-MS (Fig. 4a), the most significant increase in Fe concentration occurs when the potential drops from 0.83 to 0.74 V_RHE_. This can be explained by the Fe^3+^/Fe^2+^ redox transition at 0.76 V_RHE_ (Fig. S10, ESI†). It is worth noting that with a Fe–N–C, Fe atoms possess different formal redox and dissolution potentials depending on their coordinating ligands and extended local environment (number and size of graphene sheets,^81^ oxygen functional groups^24,28^). This broad Fe^3+^/Fe^2+^ redox range is also initially observed in Fig. S10 (ESI†). Moreover, after the increases of Fe concentration during cathodic potential shifts, gradual declines in the Fe concentration are frequently observed. This is related to the fact that the location of the Fe within the Fe–N–C structure (outer catalyst layer surface or deeper within) affects the transfer function and hence residence time. We note the high Fe dissolution could lead to problems in PEMFCs owing to the Fe species accelerating Fenton's reactions, creating hydroxyl radicals which attack the membrane, and Fe species partially exchanging with protons in the ionomer.^27,82,83^ It has been previously suggested that O-containing groups on the carbon surface reduce the turnover frequency of Fe–N–Cs by weakening O_2_-binding on FeN_*x*_ sites.^24^ The limited change in XPS O 1s spectra between pristine and 20 °C O_2_ GDE protocol (Fig. 3c) suggests performance degradation from 20 °C O_2_ protocol (Fig. 3a) is mainly attributed to active site demetallation. Meanwhile, the increase in O1s after 75 °C O_2_ protocol (Fig. 3c) causes reductions in TOF and FeN_*x*_ sites’ stability^20^ and the increased observation degradation. Reduction in TOF occurs due to reactive oxygen species catalyzing mild carbon corrosion.^24,37^

The rapid decay in O_2_ reduction performance (*e.g.* −50 ± 30 mV at 50 mA cm^−2^_geo_ after 200 cycle AST in 20 °C O_2_) and high Fe dissolution can be attributed to the high percentage of unstable high spin Fe^3+^N_*x*_ present (assuming the same type of sites are present between TAP 900@^57^Fe and TAP 900@Fe). Additionally, according to density functional theory (DFT) calculations for Fe–N–C, the number and size of graphene sheets affects the Fe dissolution potential.^81^ Previous Raman analysis of TAP 900 determined an atomically thin carbon structure,^76^ which therefore leads itself to possess less stable FeN_*x*_ sites.

The online GDE ICP-MS concentration profile under 20 °C O_2_ (Fig. 4a and c) suggests Fe dissolution and subsequent detection by ICP-MS at low current density (−0.05 to −1.00 mA cm^−2^_geo_). Meanwhile, at higher current density (−15, −50 and −100 mA cm^−2^_geo_), a process of Fe dissolution and redeposition locally into Fe_*x*_O_*y*_ in the catalyst layer is proposed. This is supported by the increased observation of Fe_*x*_O_*y*_ after O_2_ GDE protocol from HAADF-STEM and EDXS (Fig. 3f and Fig. S15, ESI†). The reason for Fe_*x*_O_*y*_ formation is hypothesized to arise based on the Fe Pourbaix diagram, where an increase in the local pH would form Fe_2_O_3_. This pH increase in the catalyst layer could occur due to the rapid consumption of H^+^ during increased O_2_ reduction currents (4H^+^ + O_2_ + 4e^−^ → 2H_2_O). It is then expected that some Fe_*x*_O_*y*_ redissolves when returning anodically to low O_2_ reduction current density (−1 mA cm^−2^_geo_), due to a return to acidic pH. This redissolution is evidenced by the detected increase in Fe concentration at −1 mA cm^−2^_geo_ on the anodic step for 20 °C O_2_ in GDE ICP-MS. The observation of Fe_*x*_O_*y*_ corroborates previous findings from *post-mortem* O_2_ AST protocols.^37,38^ Moreover, the increased Fe concentration detected when stepping the potential down in the cathodic direction after post AST (Fig. 4c) for O_2_ GDE at 20 °C and 75 °C supports the hypothesis that Fe_*x*_O_*y*_ builds up in the catalyst layer at current densities of −50 mA cm^−2^_geo_ during the AST and is only released at lower current density holds (−1 mA cm^−2^_geo_ at 20 °C and 75 °C).

We previously reported the extensive characterisation of as-prepared TAP 900@Fe, confirming the purely atomic dispersion as a pristine powder (XAS, cryo ^57^Fe Mössbauer and HAADF-STEM).^44^ A clear average change in oxidation and coordination is observed from TAP 900@Fe powder to electrode. Therefore, it appears sub-nanometric/small Fe clusters (<2 nm) form during electrode preparation (Fig. 3 and Fig. S15, S16, ESI†). This matches the recent report of Saveleva *et al.* who found, based on XAS, the ink preparation of the electrode can lead to significant changes in Fe in Fe–N–Cs from the catalyst powder to the electrode.^43^

We note that the similar XANES signals of TAP 900@Fe ink (Fig. S19, ESI†) and post O_2_ protocol are contrary to observations from HAADF-STEM (Fig. 3). This can be explained by the different probing regions of the techniques, with XANES examining the bulk electrode, where local deviations of low concentration large particles can remain hidden, which can be resolved by HAADF-STEM.

Meanwhile, the negative shift of the lower rising edge position in XANES from post 20 °C Ar protocol (Fig. S19, ESI†) is likely due to significant dissolution of Fe species with higher oxidation state, or a change in average bond length and/or coordination change.

Mass transport (O_2_ solubility and H^+^) and the thermodynamics and kinetics of ORR and Fe dissolution (at a constant potential on the RHE scale) will all change with temperature.^84^ This makes it challenging to deconvolute their contributions to changes in performance; however, kinetic modelling based on experimental data can help explain phenomena, such as local pH changes.

### Kinetic modelling

**Fig. 5:**
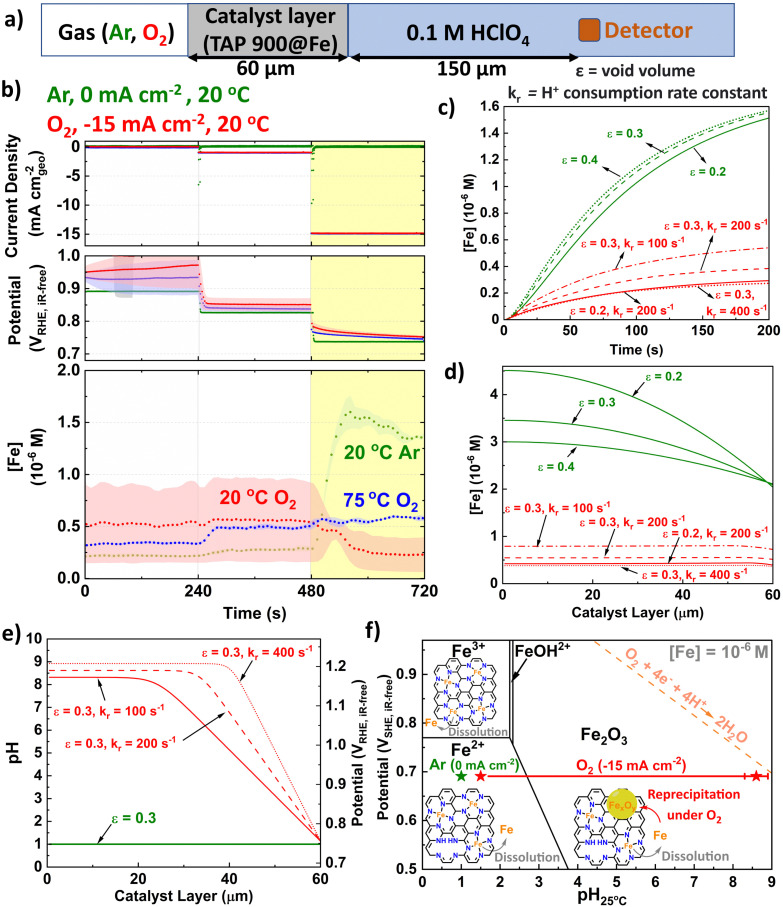
(a) Schematic depicting kinetically modelled system. (b) Initial GDE-ICP-MS data in terms of [Fe]. Simulated Fe concentration over (c) Time and (d) Catalyst layer. (e) pH and subsequent potential *vs*. RHE distribution across catalyst layer (assuming Nernstian potential shift with pH). *k*_r_ is the proton consumption rate constant and *ε* is the void volume. (f) Pourbaix diagram of Fe surface at 25 °C and [Fe] = 10^−6^ M with labelled points under O_2_ (−15 mA cm^−2^_geo_) and Ar (0 mA cm^−2^_geo_) conditions. The error bar for O_2_ represents variation of pH from different *ε* and *k*_r_ in Fig. 5e. Fe Pourbaix diagram replotted from ref. 87.

We developed a microkinetic model of the system (Fig. 5a and ESI†) to replicate the observations from GDE ICP-MS prior to AST at 20 °C in 0.1 M HClO_4_ and evidence our hypothesis on the pivotal role of local pH. The model assumed the initial proton concentration and potential in the catalyst layer is homogeneous. We focus on the Fe concentration observed in GDE ICP-MS at 0.75 V_RHE,iR-free_ and 20 °C, corresponding to a current density of −15 and 0 mA cm^−2^_geo_ under O_2_ and Ar supply, respectively. The void volume (*ε*) in the catalyst layer was adjusted to semi-quantitatively simulate the time evolution of the Fe concentration signal monitored by GDE ICP-MS in Ar-saturated electrolyte (Fig. 5b and c). The value of the proton consumption rate constant (*k*_r_) and *ε* were then varied to replicate the Fe concentration signal measured in O_2_-saturated electrolyte (Fig. 5b–d). Good agreement between experiment and simulation are reached for the range of values considered (0.2 ≤ *ε* ≤ 0.4 and 100 ≤ *k*_r_ ≤ 400 s^−1^). Additionally, values for the tortuosity factor, *τ* (=1/√*ε*) were within previously reported ranges (1.8 ≤ *τ* ≤ 2.2).^85,86^

Fig. 5e displays the corresponding simulated pH profile in the catalyst layer. The simulations predict a significantly lower concentration of detected Fe cations during O_2_ reduction (Fig. 5d). This phenomenon is attributed to the precipitation of Fe^3+^ cations under the local conditions in the catalyst layer, with the Fe concentration resulting from the balance between Fe precipitation and redissolution. Indeed, simulations indicate that at −15 mA cm^−2^_geo_, the local pH at the interface between the Fe–N–C layer and the electrolyte solution is approximately 1.5 (Fig. 5e). There is then a substantial and rapid increase in pH moving into the bulk catalyst layer (far from the liquid electrolyte), reaching pH values *ca.* 8–9. We note that the effect of Ca contamination (Fig. S16, ESI†) would be minor since we do not have evidence of local pH change under Ar and highlight that the presence of Ca^2+^ would be expected to decrease the pH rather than increase the pH (*K*s = 6.46 × 10^−6^).^88^

In terms of potential, the pH increasing to 8–9 is equivalent to *ca.* 1.1 V (Fig. 5e), based on Nernstian shift. Consequently, only the region of the catalyst layer near the solution contributes to O_2_ reduction, implying Fe–N–C thickness should be minimised, as previously proposed by Litster and coworkers from PEMFC modelling for maximised power (<30 μm).^11^ We therefore propose under practical operating current it is not possible to synchronize the pH across the catalyst layer, due to proton consumption. This supports the results of Banham *et al.* who found improved stability based on lower equivalent weight ionomer, which have increased ionic conductance.^41^ We also note from their qualitative reaction zone scheme that Fe–N–C degradation would not be as severe under air due to reduced O_2_ concentrations^41^ and therefore reduced proton consumption.

Aside from minimising Fe–N–C electrode thickness, catalyst structure modifications can be made, based on bio-inspiration.^89^ For instance, the enzyme-inspired architecture proposed by Xia *et al.*^90^ where protons and electrons are transported to the active site *via* ordered proton-conducting and electron-transporting channels. This is similar to the proposal of Middelman,^91^ who suggested the controlled self-assembly of electrodes. Methods can be adapted from the battery community who use thick electrodes (>100 μm),^92^ such as ice templating and graded electrodes.

Considering high loading Fe–N–C (thick) cathodes, one can envisage that the rate of degradation observed decreasing, as more Fe–N–C is available to react as the reaction zone passes across the degrading catalyst layer (from the membrane to backside of the Fe–N–C). Meanwhile, the intrinsic stability number^93^ of the FeN_*x*_ sites (ratio between the number of moles of Fe ions dissolved and the number of moles of oxygen being reacted) should remain the same, all else being equal.

The conditions on the Fe Pourbaix diagram are depicted in Fig. 5f. For the parameter values considered, >90% of the Fe–N–C layer experiences pH > 2.4, which would result in formation of Fe_2_O_3_ at ∼0.7 V_SHE_, for an Fe surface. Pourbaix diagrams are dependent on temperature and concentration of species. While it appears [Fe] is in order of 10^−6^ M, the Pourbaix diagram for [Fe] = 10^−8^ M at 25 °C can be found for comparison in Fig. S21 (ESI†). Additionally, Pourbaix diagrams were developed from metal surfaces rather than single atoms, where DFT-based models have instead been developed,^81^ although here it is appears the Fe–N–C system is sufficiently represented by the Pourbaix diagram of an Fe surface.^87^

## Conclusions

Online flow cell and GDE ICP-MS setup monitored the Fe concentration profile of an Fe–N–C under inert (Ar) or active (O_2_) conditions in 0.1 M HClO_4_, with temperature effects (20 and 75 °C) investigated in online GDE ICP-MS. A microkinetic model adequately represented experimental conditions in the GDE ICP-MS system with 60 μm Fe–N–C catalyst layer at 20 °C in Ar and O_2_ (at −15 mA cm^−2^_geo_). The model demonstrated a significant pH increase within the Fe–N–C layer under O_2_ at −15 mA cm^−2^_geo_ (20 °C), leading to the formation of Fe_*x*_O_*y*_ species, as confirmed from *post-mortem* characterization. Enhanced mass transport at 75 °C under O_2_ supply resulted in higher overall Fe concentration detected by GDE ICP-MS. Additionally, Fe_*x*_O_*y*_ were not detected *post-mortem* which can be due to changes in Fe Pourbaix with temperature, increased Fe redissolution at intermediate current density (−1 to −15 mA cm^−2^_geo_) and improved proton transport. Future work will explore further kinetic modelling of the 75 °C system. We propose that increased Fe concentration under both Ar- and O_2_-saturated conditions in flow cell ICP-MS arises from a lack of pH change from the limited current density (∼−1 mA cm^−2^_geo_) and therefore, low H^+^ consumption. The pH change derived from online GDE ICP-MS provides the following insights:

1. The fate of Fe (and other metal species) can vary through-plane of the catalyst layer. Therefore, the heterogeneity of Fe–N–C degradation should be considered when conducting *operando* and *post-mortem* studies.

2. Precious metal-free layers in PEMFCs, which typically employ 60–100 μm_M-N-C_ thick cathodes,^7^ may not utilize the majority of the catalyst layer during O_2_ reduction due to proton consumption. Focus, therefore, should be made on decreasing the electrode thickness by further increasing the electrochemically accessible volumetric active site density of precious metal-free catalysts. This could be facilitated by ink and ionomer optimisation (higher conductance, less bulky ionomer and tuning the ionomer:catalyst ratio), as well as designing more accessible and ordered catalyst pore structures.

## Author contributions

A. P. wrote the initial draft. A. P and J. B. synthesized the catalyst. A. P measured X-ray diffractograms and Raman spectra. J. B. measured X-ray photoelectron spectra. A. P. and V. M. measured flow cell coupled ICP-MS. A. P. and K. T. S. conducted RDE measurements and K. T. S. carried out ASTs for *post-mortem* TEM, ICP-MS and XANES. K. T. S. and V. P. measured X-ray absorption spectroscopy and V. A. S. analyzed the results. A. B. conducted microkinetic modelling. K. K. and Y.-P. K. carried out GDE coupled ICP-MS measurements. L. D. and A. H. conducted TEM analyses. L. D. and X. L. measured electron energy loss spectroscopy. S. C., I. E. L. S., M.-M. T and F. M. provided supervision and funding and revised and edited the final manuscript. A. P., and F. M. provided conceptualization.

## Data availability

Data for this article, including on-line ICP-MS, gas-diffusion electrode, X-ray photoelectron spectroscopy and microkinetic modelling, are available at Zenodo at https://doi.org/10.5281/zenodo.12742294.

## Conflicts of interest

There are no conflicts to declare.

## Supplementary Material

EE-017-D4EE01995D-s001

## References

[cit1] Luo F., Roy A., Sougrati M. T., Khan A., Cullen D. A., Wang X., Primbs M., Zitolo A., Jaouen F., Strasser P. (2023). J. Am. Chem. Soc..

[cit2] Xie X., He C., Li B., He Y., Cullen D. A., Wegener E. C., Kropf A. J., Martinez U., Cheng Y., Engelhard M. H., Bowden M. E., Song M., Lemmon T., Li X. S., Nie Z., Liu J., Myers D. J., Zelenay P., Wang G., Wu G., Ramani V., Shao Y. (2020). Nat. Catal..

[cit3] Chen M., Li X., Yang F., Li B., Stracensky T., Karakalos S., Mukerjee S., Jia Q., Su D., Wang G., Wu G., Xu H. (2020). ACS Catal..

[cit4] Luo F., Roy A., Silvioli L., Cullen D. A., Zitolo A., Sougrati M. T., Oguz I. C., Mineva T., Teschner D., Wagner S., Wen J., Dionigi F., Kramm U. I., Rossmeisl J., Jaouen F., Strasser P. (2020). Nat. Mater..

[cit5] Jiao L., Li J., Richard L. L., Sun Q., Stracensky T., Liu E., Sougrati M. T., Zhao Z., Yang F., Zhong S., Xu H., Mukerjee S., Huang Y., Cullen D. A., Park J. H., Ferrandon M., Myers D. J., Jaouen F., Jia Q. (2021). Nat. Mater..

[cit6] Liu S., Li C., Zachman M. J., Zeng Y., Yu H., Li B., Wang M., Braaten J., Liu J., Meyer H. M., Lucero M., Kropf A. J., Alp E. E., Gong Q., Shi Q., Feng Z., Xu H., Wang G., Myers D. J., Xie J., Cullen D. A., Litster S., Wu G. (2022). Nat. Energy.

[cit7] Banham D., Choi J. Y., Kishimoto T., Ye S. (2019). Adv. Mater..

[cit8] Gasteiger H. A., Kocha S. S., Sompalli B., Wagner F. T. (2005). Appl. Catal., B.

[cit9] Mehmood A., Gong M., Jaouen F., Roy A., Zitolo A., Khan A., Sougrati M., Primbs M., Bonastre A. M., Fongalland D., Drazic G., Strasser P., Kucernak A. (2022). Nat. Catal..

[cit10] Jaouen F., Jones D., Coutard N., Artero V., Strasser P., Kucernak A. (2018). Johnson Matthey Technol. Rev..

[cit11] Babu S. K., Chung H. T., Zelenay P., Litster S. (2017). J. Electrochem. Soc..

[cit12] Thompson S. T., Papageorgopoulos D. (2019). Nat. Catal..

[cit13] Pedersen A., Pandya J., Leonzio G., Serov A., Bernardi A., Stephens I., Titirici M.-M., Petit C., Chachuat B. (2023). Green Chem..

[cit14] Pedersen A., Bagger A., Barrio J., Maillard F., Stephens I., Titirici M.-M. (2023). J. Mater. Chem. A.

[cit15] Kumar K., Gairola P., Lions M., Ranjbar-Sahraie N., Mermoux M., Dubau L., Zitolo A., Jaouen F., Maillard F. (2018). ACS Catal..

[cit16] Zeng Y., Li C., Li B., Liang J., Zachman M. J., Cullen D. A., Hermann R. P., Alp E. E., Lavina B., Karakalos S., Lucero M., Zhang B., Wang M., Feng Z., Wang G., Xie J., Myers D. J., Dodelet J.-P., Wu G. (2023). Nat. Catal..

[cit17] US DOE - Hydrogen and Fuel Cell Technologies Office, Hydrogen and Fuel Cell Technologies Office Multi-Year Research, Development, and Demonstration Plan. Section 3.4: Fuel Cells, 2017

[cit18] Kumar K., Dubau L., Jaouen F., Maillard F. (2023). Chem. Rev..

[cit19] Choi C. H., Baldizzone C., Grote J.-P., Schuppert A. K., Jaouen F., Mayrhofer K. J. J. (2015). Angew. Chem., Int. Ed..

[cit20] Tan X., Tahini H. A., Smith S. C. (2021). J. Mater. Chem. A.

[cit21] Herranz J., Jaouen F., Lefèvre M., Kramm U. I., Proietti E., Dodelet J. P., Bogdanoff P., Fiechter S., Abs-Wurmbach I., Bertrand P., Arruda T. M., Mukerjee S. (2011). J. Phys. Chem. C.

[cit22] Kumar K., Asset T., Li X., Liu Y., Yan X., Chen Y., Mermoux M., Pan X., Atanassov P., Maillard F., Dubau L. (2021). ACS Catal..

[cit23] Chenitz R., Kramm U. I., Lefèvre M., Glibin V., Zhang G., Sun S., Dodelet J.-P. (2018). Energy Environ. Sci..

[cit24] Choi C. H., Lim H. K., Chung M. W., Chon G., Ranjbar Sahraie N., Altin A., Sougrati M. T., Stievano L., Oh H. S., Park E. S., Luo F., Strasser P., Dražić G., Mayrhofer K. J. J., Kim H., Jaouen F. (2018). Energy Environ. Sci..

[cit25] Ünsal S., Girod R., Appel C., Karpov D., Mermoux M., Maillard F., Saveleva V. A., Tileli V., Schmidt T. J., Herranz J. (2023). J. Am. Chem. Soc..

[cit26] Bae G., Kim M. M., Han M. H., Cho J., Kim D. H., Sougrati M.-T., Kim J., Lee K.-S., Joo S. H., Goddard W. A., Oh H.-S., Kim H., Jaouen F., Choi C. H. (2023). Nat. Catal..

[cit27] Choi C. H., Baldizzone C., Polymeros G., Pizzutilo E., Kasian O., Schuppert A. K., Ranjbar Sahraie N., Sougrati M.-T., Mayrhofer K. J. J., Jaouen F. (2016). ACS Catal..

[cit28] Boldrin P., Malko D., Mehmood A., Kramm U. I., Wagner S., Paul S., Weidler N., Kucernak A. (2021). Appl. Catal., B.

[cit29] Shao Y., Dodelet J., Wu G., Zelenay P. (2019). Adv. Mater..

[cit30] Zhang H., Osmieri L., Park J. H., Chung H. T., Cullen D. A., Neyerlin K. C., Myers D. J., Zelenay P. (2022). Nat. Catal..

[cit31] Ferrandon M., Wang X., Kropf A. J., Myers D. J., Wu G., Johnston C. M., Zelenay P. (2013). Electrochim. Acta.

[cit32] Liu S., Meyer Q., Jia C., Wang S., Rong C., Nie Y., Zhao C. (2023). Energy Environ. Sci..

[cit33] Santori P. G., Speck F. D., Li J., Zitolo A., Jia Q., Mukerjee S., Cherevko S., Jaouen F. (2019). J. Electrochem. Soc..

[cit34] Lopes P. P. (2023). ACS Mater. Au.

[cit35] Ehelebe K., Knöppel J., Bierling M., Mayerhöfer B., Böhm T., Kulyk N., Thiele S., Mayrhofer K. J. J., Cherevko S. (2021). Angew. Chem., Int. Ed..

[cit36] Nikkuni F. R., Vion-Dury B., Dubau L., Maillard F., Ticianelli E. A., Chatenet M. (2014). Appl. Catal., B.

[cit37] Kumar K., Dubau L., Mermoux M., Li J., Zitolo A., Nelayah J., Jaouen F., Maillard F. (2020). Angew. Chem., Int. Ed..

[cit38] Li J., Sougrati M. T., Zitolo A., Ablett J. M., Oğuz I. C., Mineva T., Matanovic I., Atanassov P., Huang Y., Zenyuk I., Di Cicco A., Kumar K., Dubau L., Maillard F., Dražić G., Jaouen F. (2021). Nat. Catal..

[cit39] Goellner V., Baldizzone C., Schuppert A., Sougrati M. T., Mayrhofer K., Jaouen F. (2014). Phys. Chem. Chem. Phys..

[cit40] Osmieri L., Cullen D. A., Chung H. T., Ahluwalia R. K., Neyerlin K. C. (2020). Nano Energy.

[cit41] Banham D., Kishimoto T., Sato T., Kobayashi Y., Narizuka K., Ichi Ozaki J., Zhou Y., Marquez E., Bai K., Ye S. (2017). J. Power Sources.

[cit42] Santos K. T., Kumar K., Dubau L., Ge H., Berthon-Fabry S., Vasconcellos C. S. A., Lima F. H. B., Asset T., Atanassov P., Saveleva V. A., Glatzel P., Li X., Jaouen F., Maillard F. (2023). J. Power Sources.

[cit43] Saveleva V. A., Kumar K., Theis P., Salas N. S., Kramm U. I., Jaouen F., Maillard F., Glatzel P. (2023). ACS Appl. Energy Mater..

[cit44] Barrio J., Pedersen A., Sarma S. Ch, Bagger A., Gong M., Favero S., Zhao C., Garcia-Serres R., Li A. Y., Zhang Q., Jaouen F., Maillard F., Kucernak A., Stephens I. E. L., Titirici M. (2023). Adv. Mater..

[cit45] Primbs M., Sun Y., Roy A., Malko D., Mehmood A., Sougrati M.-T., Blanchard P.-Y., Granozzi G., Kosmala T., Daniel G., Atanassov P., Sharman J., Durante C., Kucernak A., Jones D., Jaouen F., Strasser P. (2020). Energy Environ. Sci..

[cit46] Wan X., Liu X., Li Y., Yu R., Zheng L., Yan W., Wang H., Xu M., Shui J. (2019). Nat. Catal..

[cit47] Zhong J.-Q., Yan K.-J., Yang J., Yang W.-H., Yang X.-D. (2022). ACS Catal..

[cit48] Jaouen F., Goellner V., Lefèvre M., Herranz J., Proietti E., Dodelet J. P. (2013). Electrochim. Acta.

[cit49] Gridin V., Du J., Haller S., Theis P., Hofmann K., Wiberg G. K. H., Kramm U. I., Arenz M. (2023). Electrochim. Acta.

[cit50] Malko D., Kucernak A., Lopes T. (2016). Nat. Commun..

[cit51] Rojas-Carbonell S., Artyushkova K., Serov A., Santoro C., Matanovic I., Atanassov P. (2018). ACS Catal..

[cit52] Gong M., Mehmood A., Ali B., Nam K.-W., Kucernak A. (2023). ACS Catal..

[cit53] Bonnefont A. (2023). Curr. Opin. Electrochem..

[cit54] Varela A. S., Kroschel M., Reier T., Strasser P. (2016). Catal. Today.

[cit55] Varela A. S. (2020). Curr. Opin. Green Sustainable Chem..

[cit56] Muthukrishnan A., James A. (2022). Catal. Sci. Technol..

[cit57] Bae G., Chung M. W., Ji S. G., Jaouen F., Choi C. H. (2020). ACS Catal..

[cit58] Rouhet M., Bozdech S., Bonnefont A., Savinova E. R. (2013). Electrochem. Commun..

[cit59] Monteiro M. C. O., Koper M. T. M. (2021). Curr. Opin. Electrochem..

[cit60] Sauvé E. R., Tang B. Y., Razdan N. K., Toh W. L., Weng S., Surendranath Y. (2024). Joule.

[cit61] Zenyuk I. V., Litster S. (2013). ECS Trans..

[cit62] Jaouen F., Lefèvre M., Dodelet J.-P., Cai M. (2006). J. Phys. Chem. B.

[cit63] Choi J.-Y., Yang L., Kishimoto T., Fu X., Ye S., Chen Z., Banham D. (2017). Energy Environ. Sci..

[cit64] Yin X., Zelenay P. (2018). ECS Trans..

[cit65] Zhang G., Yang X., Dubois M., Herraiz M., Chenitz R., Lefèvre M., Cherif M., Vidal F., Glibin V. P., Sun S., Dodelet J.-P. (2019). Energy Environ. Sci..

[cit66] Dodelet J.-P., Glibin V., Zhang G., Kramm U. I., Chenitz R., Vidal F., Sun S., Dubois M. (2021). Energy Environ. Sci..

[cit67] Yin X., Holby E. F., Zelenay P. (2021). Energy Environ. Sci..

[cit68] Pedersen A., Snitkoff-Sol R. Z., Presman Y., Barrio J., Cai R., Suter T., Yang G., Haigh S. J., Brett D., Jervis R., Titirici M.-M., Stephens I. E. L., Elbaz L. (2024). J. Power Sources.

[cit69] Ehelebe K., Seeberger D., Paul M. T. Y., Thiele S., Mayrhofer K. J. J., Cherevko S. (2019). J. Electrochem. Soc..

[cit70] Ku Y.-P., Ehelebe K., Hutzler A., Bierling M., Böhm T., Zitolo A., Vorokhta M., Bibent N., Speck F. D., Seeberger D., Khalakhan I., Mayrhofer K. J. J., Thiele S., Jaouen F., Cherevko S. (2022). J. Am. Chem. Soc..

[cit71] Gayer K. H., Woontner L. (1956). J. Phys. Chem..

[cit72] Pourbaix M. , Atlas of Electrochemical Equilibria in Aqueous Solutions, National Association of Corrosion Engineers, 1974

[cit73] Ebner K., Ni L., Saveleva V. A., Monnier B. P. L., Clark A. H., Krumeich F., Nachtegaal M., Luterbacher J. S., Kramm U. I., Schmidt T. J., Herranz J. (2021). Phys. Chem. Chem. Phys..

[cit74] Yue G., Zhao L., Olvera O. G., Asselin E. (2014). Hydrometallurgy.

[cit75] Charlot G. , Les méthodes de la chimie analytique: analyse quantitative minérale, Masson, 1961

[cit76] Sarma S. C., Barrio J., Bagger A., Pedersen A., Gong M., Luo H., Wang M., Favero S., Zhao C., Zhang Q., Kucernak A., Titirici M., Stephens I. E. L. (2023). Adv. Funct. Mater..

[cit77] Ramaswamy N., Tylus U., Jia Q., Mukerjee S. (2013). J. Am. Chem. Soc..

[cit78] Wang X., Ferrandon M., Park J. H., Shen J.-J., Kropf A. J., Zhang H., Zelenay P., Myers D. J. (2023). Electrochim. Acta.

[cit79] Paulus U. A., Schmidt T. J., Gasteiger H. A., Behm R. J. (2001). J. Electroanal. Chem..

[cit80] Jackson M. N., Jung O., Lamotte H. C., Surendranath Y. (2019). ACS Catal..

[cit81] Holby E. F., Wang G., Zelenay P. (2020). ACS Catal..

[cit82] Pozio A., Silva R. F., De Francesco M., Giorgi L. (2003). Electrochim. Acta.

[cit83] Goellner V., Armel V., Zitolo A., Fonda E., Jaouen F. (2015). J. Electrochem. Soc..

[cit84] Neyerlin K. C., Gu W., Jorne J., Gasteiger H. A. (2006). J. Electrochem. Soc..

[cit85] Komini Babu S., Chung H. T., Zelenay P., Litster S. (2016). ACS Appl. Mater. Interfaces.

[cit86] Ridge S. J., White R. E., Tsou Y., Beaver R. N., Eisman G. A. (1989). J. Electrochem. Soc..

[cit87] Beverskog B., Puigdomenech I. (1996). Corros. Sci..

[cit88] Martell A. E. and Smith R. M., Critical Stability Constants: Inorganic Complexes, Plenum Press, 1976

[cit89] Barrio J., Pedersen A., Favero S., Luo H., Wang M., Sarma S. Ch, Feng J., Ngoc L. T. T., Kellner S., Li A. Y., Jorge Sobrido A. B., Titirici M.-M. (2023). Chem. Rev..

[cit90] Xia Z., Wang S., Jiang L., Sun H., Liu S., Fu X., Zhang B., Sheng Su D., Wang J., Sun G. (2015). Sci. Rep..

[cit91] Middelman E. (2002). Fuel Cells Bull..

[cit92] Boyce A. M., Cumming D. J., Huang C., Zankowski S. P., Grant P. S., Brett D. J. L., Shearing P. R. (2021). ACS Nano.

[cit93] Geiger S., Kasian O., Ledendecker M., Pizzutilo E., Mingers A. M., Fu W. T., Diaz-Morales O., Li Z., Oellers T., Fruchter L., Ludwig A., Mayrhofer K. J. J., Koper M. T. M., Cherevko S. (2018). Nat. Catal..

