# Supplementary material for: Operando Fe dissolution in Fe–N–C electrocatalysts during acidic oxygen reduction: impact of local pH change
Source: Energy Environ Sci. 2024 Jul 30;17(17):6323–37. doi: 10.1039/d4ee01995d (PMC11348952; doi:10.1039/d4ee01995d)
Supplement: EE-017-D4EE01995D-s001 [file EE-017-D4EE01995D-s001.pdf]

Supplementary Information (SI) for Energy & Environmental Science.
This journal is © The Royal Society of Chemistry 2024

Supporting Information:

Operando Fe Dissolution in Fe-N-C Electrocatalysts during Acidic Oxygen Reduction: Impact of Local pH Change

Angus Pedersen,[a,b,c‡] Kavita Kumar,[d‡] Yu-Ping Ku,[d,e] Vincent Martin,[c] Laetitia Dubau,[c] Keyla Teixeira Santos,[c] Jesús Barrio,[a,b] Viktoriia A. Saveleva,[f] Pieter Glatzel,[f] Vinod K. Paidi,[f] Xiaoyan Li,[g] Andreas Hutzler,[d] Maria-Magdalena Titirici,[b] Antoine Bonnefont,[c] Serhiy Cherevko,[d] Ifan E. L. Stephens,[a,*] Frédéric Maillard[c] *

[a] *Imperial College London, Department of Materials, Royal School of Mines, London SW7 2AZ, UK*

[b] *Imperial College London, Department of Chemical Engineering, London SW7 2AZ, UK*

[c] *Univ. Grenoble Alpes, Univ. Savoie-Mont-Blanc, CNRS, Grenoble-INP, LEPMI, 38000 Grenoble, France*

[d] *Forschungszentrum Jülich GmbH, Helmholtz-Institute Erlangen-Nürnberg for Renewable Energy (HI ERN), Cauerstraße 1, 91058 Erlangen, Germany*

[e] *Friedrich-Alexander-Universität Erlangen-Nürnberg, Department of Chemical and Biological Engineering, Cauerstraße 1, 91058 Erlangen, Germany*

[f] *ESRF, The European Synchrotron, 71 Avenue des Martyrs, CS40220, 38043 Grenoble Cedex 9, France*

[g] *Laboratoire de Physique des Solides CNRS, Université Paris Sud, 91405 Orsay, France*

*Corresponding authors:

frederic.maillard@grenoble-inp.fr

i.stephens@imperial.ac.uk

Table of Contents

1. Experimental Details 3
1.1 Synthesis 3
1.2 *Operando* Characterisation 3
1.3 *Ex Situ* Characterisation 5
2. Equations 7
2.1 Kinetic Modelling 7
3. Results 9
References 31

1. Experimental Details

1.1 Synthesis

TAP 900@Fe: 2,4,6-triaminopyrimidine (97%, Sigma Aldrich) and $MgCl_2 \cdot 6H_2O$ (99%, Sigma Aldrich) were ground together with a pestle and mortar in a respective 1:8 weight ratio and then pyrolyzed in a ceramic crucible at 5 °C min^{-1} up to 900 °C and then one hour hold, all under 300 mL min^{-1} flow of N_2 atmosphere (>99.998%, BOC). The resultant materials were ground to fine powder with a pestle and mortar, and then washed with 2 M HCl (prepared by dilution of fuming 37% HCl, Merck) overnight. Next, the solution was filtered, rinsed thoroughly with distilled water, and dried at 80 °C overnight, forming TAP 900.

Fe was impregnated in TAP 900 via a low-temperature methanol reflux as previously reported.[1–3] 60 mg of TAP 900 was placed in a 250 mL round-bottom flask along with 75 mL MeOH (AnalaR NORMAPUR Reag. Ph. Eur., ACS, VWR) under vigorous stirring until a homogeneous dispersion was observed. Then, 75 mL of MeOH solution containing 25×10^{-3} M $FeCl_2$ (98% Sigma Aldrich) was added, and the solution was refluxed at 85-90 °C for 24 h. The product was then filtered and rinsed with MeOH, and the powder washed with 0.5 M H_2SO_4 (95-98% Sigma Aldrich) overnight. Finally, the obtained material was filtered and rinsed abundantly with distilled water and dried at 80 °C to obtain TAP 900@Fe.

TAP 900@^{57}Fe: For TAP 900@^{57}Fe, the iron precursor iron chloride $^{57}FeCl_2$ was first prepared. ^{57}Fe (250 mg, Iron-57, 99.9% purity with 95.5% ^{57}Fe, Cambridge Isotope Laboratories) was added into a 1:1 37% HCl (Analytical reagent grade (>99.99%), Fisher Chemical) and deionised H_2O solution (50 mL), then reflux (115°C) was performed for 3h until everything was dissolved. Finally, $^{57}FeCl_2$ was obtained after leaving solution evaporating under vacuum at 60 °C for 3 hours, then 80°C overnight and freeze drying overnight.

FeNC samples and their electrodes were stored under O_2-free environments (vacuum or N_2/Ar boxes) to prevent spontaneous aerobic ageing of the FeNC.[4]

1.2 *Operando* Characterisation

Flow Cell and Rotating Disc Electrode Preparation: consisted of a 5 mm glassy carbon disc (SIGRADUR, HTW GmbH) in a homemade PEEK shroud. RDE tips were cleaned in Caro acid (prepared by mixing in 1:1 ratio of 30% H_2O_2 with 96% H_2SO_4) for at least 5 hours. The RDE tips were then boiled in Milli-Q water twice and placed in an oven at 60°C to remove remaining water. The RDE tip was then polished with 3 µm and then 1 µm diamond paste (Presi) on a polishing pad (Presi) to a mirror finish.

RDE Electrode Manufacture: Catalyst inks for RDE and flow cell comprised of 4 mg_{FeNC} mL^{-1}, consisting of a ratio of 4 mg TAP 900@^{57}Fe, 480 µL of 18.2 MΩ cm^{-1} deionized (Milli-Q) H_2O, 480 µL of isopropanol (Fisher, 99.5 %) and 40 µL of 5 wt.% Nafion® (5% w/w in water and 1-propanol, Sigma Aldrich). The ink was bath sonicated (Fisherbrand FB 15046, 30 W, 50/60 Hz) for 30 mins and left overnight. The ink was bath sonicated again for 1 min prior to drop casting. Inks were drop-cast onto the glassy carbon surface to the desired loading and subsequently dried under rotation at 100-200 rpm with a heat gun parallel to the RDE tip.

Due to the hydrophobicity of the catalyst, a droplet of electrolyte was placed over the RDE tip and placed in a desiccator and pumped down to vacuum. Upon removal, the catalyst was wet,

and all visible air bubbles were removed. With the droplet remaining on the catalyst, the RDE tip was placed in the homemade flow cell (**Figure S1**) or RDE cell.

Flow Cell ICP-MS Protocol: All measurements were conducted at room temperature (21 ± 1 ^{o}C). Ar or O_2 was purged in the feed electrolyte (0.1 M $HClO_4$ or 0.05 M H_2SO_4) for at least 20 mins prior to measurements. The potential was held at 0.9 V_{RHE} for 200 s before and after each measurement to confirm stable background ICP-MS measurement. Ohmic drop (*R*) in the cell was first determined via electrochemical impedance measurement at open circuit potential (OCP) from the intercept of the real impedance axis. *R* varied from 5-25 Ω, if *R* >25 Ω the cell was reinstalled following checks for oxygen bubbles on the catalytic layer surface.

Initial 50 cyclic voltammetry (CV) under Ar-saturation between 0.925-0.200 V_{RHE} at 50 mV s^{-1}, followed by 6 CVs under Ar-saturation 0.925-0.200 V_{RHE} at 10 mV s^{-1} and 6 CVs under O_2-saturation between 0.925-0.200 V_{RHE} at 10 mV s^{-1}. Next, either accelerated stress testing (AST) under O_2-saturation was carried out for 1 h, holding the potential at 0.9 and 0.6 V_{RHE} for 3 s intervals, or chronoamperometry (CA) holding at 0.2 or 0.6 V_{RHE} for 1 h under O_2-saturation. The square wave AST and its potentials mimics the protocol recently established for Fe-N-C-based PEMFCs.[5] For Ar AST, O_2-saturated measurements were not conducted beforehand due to some O_2 remaining in the system, which may have affected the results. Finally, 6 CVs under O_2-saturation between 0.925-0.200 V_{RHE} at 10 mV s^{-1}. A synchronization step from 0.9 to 0.2 to 0.9 V_{RHE} was applied at the end of each measurement to align ICP-MS and electrochemical data. The protocol is summarized in **Figure S2**. In the instance where slow (1 mV s^{-1}) CVs were carried out, this proceeded the 6 CVs (in Ar and O_2) and was not followed by any subsequent measurement.

Flow Cell ICP-MS Operation: Flowrate of electrolyte was monitored each day (varying from 430-480 µL min^{-1}). The ICP-MS was calibrated before and after completing the electrochemical measurement protocol. Four-point calibration curves were measured with a blank and five prepared standard solutions of ^{56}Fe and ^{24}Mg at 0, 5, 10, 20 and 50 ppb. The calibration factor of ^{57}Fe was corrected with its natural abundance of 2.12%. Calibration curves were carried out before and after online ICP-MS measurements with correction applied for drift. ^{57}Fe and ^{24}Mg were analyzed with a PerkinElmer NexION 2000c spectrometer coupled to the home-made electrochemical flow cell (**Figure S2**) and data was recorded on Syngistix software.

GDE ICP-MS Protocol: Each measurement began with 50 cycles at 50 mV s^{-1} in Ar atmosphere between 0.20 and 0.925 $V_{RHE,}$ with the electrolyte subsequently refreshed (**Figure S3**). Then, two different electrochemical techniques were used, depending on the gas atmosphere studied. Potentiostatic and galvanostatic techniques were used in Ar and O_2 atmosphere, respectively. This is due to unpredictable uncompensated resistance recorded at varied current densities in O_2 atmosphere during potentiostatic holds and the iR-compensated potential would not be comparable in the two gas conditions. To mimic recently standardized PEMFC AST conditions,[5] under O_2 conditions in GDE, the current was held for 3 s intervals 200 times at -0.05 and -50 mA cm^{-2}_{geo}, corresponding to *ca*. 0.85 and 0.6 $V_{RHE,\ iR\text{-}free}$, respectively. Under Ar, the potential was held for 3 s intervals 200 times between 0.9 and 0.6 $V_{RHE,\ iR\text{-}free}$. Before and after AST, 240 s of potentiostatic and galvanostatic holds were applied between (0.85 and 0.50 $V_{RHE,\ iR\text{-}free}$) and (-1 and -100 mA cm^{-2}_{geo}), respectively, as illustrated in **Figure S3**.

GDE ICP-MS Operation: An ICP-MS (Perkin Elmer, NexION 350) was operated in dynamic reaction cell mode with methane (99.9995%, Air Liquide) to limit the impact of polyatomic interference between ^{56}Fe and $^{40}Ar^{16}O^{+}$. 2.5 ppb of germanium (Ge) in 1 wt. % HNO_3

(Ultrapure, Merck) was used as internal standard to track and compensate for instrument drift during measurements. Five-point calibration lines were measured with a blank and four daily prepared standard solutions of Fe (0, 1, 5, 25 and 50 ppb). Between 0.203-0.226 mL min^{-1} of the electrolyte was continuously extracted via a capillary to the ICP-MS. The collection efficiency (CE) is calculated using **Equation S2**. (**Figure S3**). Calculated Fe dissolution (**Equation S3**) is based on catalyst loadings, flowrate, and collection efficiencies in **Table S1**. Fe dissolution data from GDE-ICP-MS was smoothed by adjacent-averaging 30 neighboring points.

1.3 *Ex Situ* Characterisation

RDE measurements were carried out in a three-electrode setup, employing an AUTOLAB PGSTAT302N in Ar- (99.999%, Messer) or O_2 (99.999%, Messer) saturated 0.1 M $HClO_4$ electrolyte. Measurements in 0.1 M $HClO_4$ (Suprapur, Carl Roth) consisted of initial 50 CV in Ar at 50 mV s^{-1} followed by O_2 saturated conditions at 1,600 rpm and 10 mV s^{-1} at 25°C. 85% *iR* correction was applied during O_2 reduction measurement. O_2 reduction capacitance correction was applied by subtracting current under equivalent measurement conditions, under Ar-saturation.

Raman spectra were measured with an inVia Renishaw confocal Raman microscope with a 532 nm incident laser beam focused through a 50× objective (Leica). The laser intensity was minimized to 2.6 mW to avoid laser damage to the samples.

High-Angle Annular Dark Field Scanning Transmission Electron Microscopy (HAADF-STEM) was performed with a Talos F200i (ThermoFisher Scientific) operated at an acceleration voltage of 200 kV, a beam current of *ca* 40 pA, and a convergence angle of 10.5 mrad.
STEM combined with **electron energy loss spectra** (EELS) were performed using a C_3/C_5 corrected Nion microscope USTEM-200. The experiments are done at 60 keV (to limit beam damage) with a convergence semi-angle of 33mrad. HAADF images were acquired with 80–200 mrad collection angle and the EELS were acquired using a MerlinEM direct electron detector (Quantum Detectors Ltd).

Energy Dispersive X-ray Spectroscopy (EDXS) employed a Dual Bruker XFlash 6T-100 EDS detector.

Fe Kβ **High Energy-Resolution Fluorescence-Detected X-ray Absorption Near Edge Structure** (HERFD-XANES) experiments were conducted at beamline ID26, European Synchrotron Radiation Facility (ESRF). The X-ray beam incidence was 45°, creating a bulk-sensitive XANES signal. The pristine catalyst powder and thin films of catalytic suspensions were analyzed, utilizing three undulators (u35) to produce incoming photons. A pair of cryogenically cooled Si(111) crystals then monochromatized the radiation. To calibrate the incident beam energy, a reference metallic Fe foil set the first inflection point of the Fe K edge at 7112 eV. Note that the XANES spectra of Fe foil was obtained in transmission mode, where the foil was under ultra-high vacuum conditions to preserve its metallic state. Due to the difference in the experimental conditions of Fe foil and the samples discussed here, the direct comparison of the absolute XANES intensities cannot be made. Instead, for Fe foil, focus is made on the shape of the XANES. In the experimental setup, Germanium (Ge) crystal analyzers (Ge(620) for Kβ and Ge(440) for Kα) in Rowland geometry were employed. The TAP 900@Fe powder was blended with BN3 (Aldrich®) at a mass ratio of 1:3 and subsequently compressed into pellets using a 1-ton force. This identical procedure was applied to both FeO (Aldrich®) and Fe_2O_3 (Aldrich®). These pellets, along with GDE and glassy carbon rods carrying fresh ink or *post-*

mortem catalyst samples were positioned in a sample holder.

X-Ray Diffraction (XRD) patterns were obtained using a PANanalytical X'PERT PRO powder X-ray diffractometer with a Cu Kα source operated at 40 kV and 40 mA, with 0.033^{o} scan step size.

X-ray Photoelectron Spectroscopy (XPS) was measured with a Thermo Fisher K-alpha XPS system, and the spectra analyzed with Avantage software. All spectra were calibrated relative to the carbon C1s peak at 284.8 eV to correct for charging effects. 200 scans were measured in N and O spectra. The Fe peak of TAP 900@Fe mixed with Nafion could not be resolved in XPS due to attenuation of the Fe2p signal by the fluorine Auger peak.[6] It is also noted that X-rays cause beam damage to Nafion, however since equivalent number of cycles were repeated on XPS peaks (*e.g*. O1s) for GDE samples any damage will be equivalent for all samples.[7]

2. Equations

The mass activity, m_{act}, can be found via:

$$m_{act} = \frac{j}{L_{FeNC}} \quad \text{Eq. S1}$$

Where L_{FeNC} is the mass loading of catalyst ($mg_{Fe\text{-}N\text{-}C}$ cm^{-2}_{geo}) and j is the current density (mA cm^{-2}_{geo}) at a specified potential.

For the quantification of the Fe dissolution in GDE-ICP-MS setup, the collection efficiency (CE) was extracted from **Eq. S2**:

$$CE = \frac{m_{Fe,\,ICP-MS}}{m_{Fe,\,bulk,end} - m_{Fe,bulk,\,start} + m_{Fe,\,ICP-MS}} \quad \text{Eq. S2}$$

Where $m_{Fe,ICP\text{-}MS}$ is the total Fe amount collected in the ICP-MS, $m_{Fe,bulk,start}$ and $m_{Fe,bulk,end}$ are the quantity of Fe in the bulk electrolyte before and after the measurement, respectively. The CE values obtained are presented in Table S1.

The rate of Fe dissolution is given by **Eq. S3**:[8]

$$\frac{dFe}{dt} = \frac{[Fe] \cdot Q}{m_{FeNC} \cdot A \cdot CE} \quad \text{Eq. S3}$$

Where dFe/dt is the rate of Fe dissolution normalised to catalyst loading (ng_{Fe} s^{-1} g_{FeNC}^{-1}), [Fe] is the concentration of Fe (ng_{Fe} L^{-1}), Q is flowrate (L s^{-1}), and A is the area of the RDE tip (0.19635 cm^2) or GDE electrode (2.01 cm^2). CE is the collection efficiency. CE is assumed 100% in flow cell ICP-MS and is calculated in GDE ICP-MS (see **Table S1** for values).

2.1 Kinetic Modelling

The concentrations of proton (C_H), Fe (C_{Fe}) and hydroxide ions (C_{OH}) as a function to time (t) and space (x) are modelled by solving a system of partial differential equations describing the effect of mass transport and of reactions in the catalyst layer and in the electrolyte.
In the catalyst layer:

$$\frac{\partial C_{Fe}}{\partial t} = D_{Fe,eff} \frac{\partial^2 C_{Fe}}{\partial x^2} + k_{dis}\left(1 - H\left(\frac{C_{Fe}\,Ke^3}{C_H{}^3 Ks} - 1\right)\right)$$

$$\frac{\partial C_H}{\partial t} = D_{H,eff} \frac{\partial^2 C_H}{\partial x^2} + 3k_{dis}\ H\left(\frac{C_{Fe}\,Ke^3}{C_H{}^3 Ks} - 1\right) - kr\,CH + kfw - kbw\,CH\,COH$$

$$\frac{\partial C_{OH}}{\partial t} = D_{OH,eff} \frac{\partial^2 C_{OH}}{\partial x^2} + kfw - kbw\,CH\,COH$$

In solution:

$$\frac{\partial C_{Fe}}{\partial t} = DFe\frac{\partial^2 C_{Fe}}{\partial x^2}$$

$$\frac{\partial C_H}{\partial t} = D_H\frac{\partial^2 C_H}{\partial x^2} + kfw - kbw\ CH\ COH$$

$$\frac{\partial C_{OH}}{\partial t} = D_{OH}\frac{\partial^2 C_{OH}}{\partial x^2} + kfw - kbw\ CH\ COH$$

The reactions considered in the model are:

- the self-ionization of water, $H_2O = H^+ + OH^-$ (k_{fw}=1.4 × 10^{-3} M s^{-1}, k_{bw}= 7 × 10^{10} M^{-1} s^{-1})[9]
- the Fe dissolution, k_{dis} = 8 × 10^{-11} s^{-1} in O_2 and 4 × 10^{-11} s^{-1} in Ar (applied from reproducing experimental data)
- the Fe precipitation: $Fe^{3+} + 3H_2O = Fe(OH)_3 + 3\ H^+$ (solubility constant of $Fe(OH)_3$, K_s=2.8 x10^{-39})[10]

D_{Fe} = 6 × 10^{-6} cm^2 s^{-1},[11] while D_H is estimated by using the expression for the diffusion coefficient of binary electrolyte:

$$D_H = \frac{2D_{H^+} \cdot D_{ClO_4}}{D_{H^+} + D_{ClO_4}} = 2.72 \times 10^{-5}\ cm^2\ s^{-1}$$

In the catalyst layer, the effective diffusion coefficient was calculated using the Bruggeman equation:[12]

$$D_{i,eff} = D_i \times \varepsilon^{3/2}$$

Where ε is the volume fraction of the aqueous phase in the catalyst layer.

From the Bruggeman model:

$$\tau = \frac{1}{\sqrt{\varepsilon}}$$

Where τ is the tortuosity factor.

The system of partial differential equations is solved for the initial conditions, at t = 0, C_{Fe} = 0 and C_H = 10^{-1} M, C_{OH} = 10^{-13} M. The following boundary conditions are used:
At the gas phase/catalyst layer interface at x = 0:

$$D_{Fe,eff}\frac{\partial C_{Fe}}{\partial x} = 0$$

$$D_{H,eff}\frac{\partial C_H}{\partial x} = 0$$

$$D_{OH}\frac{\partial C_{OH}}{\partial x} = 0$$

At the catalyst layer/liquid electrolyte interface at x = 60 µm:

$$D_{Fe,eff}\frac{\partial C_{Fe}}{\partial x} = D_{Fe}\frac{\partial C_{Fe}}{\partial x}$$

$$D_H\frac{\partial C_H}{\partial x} = -\frac{j}{F}$$

Where j = -15 mA cm^{-2}_{geo} is the faradaic current density under O_2 reduction conditions.

3. Results

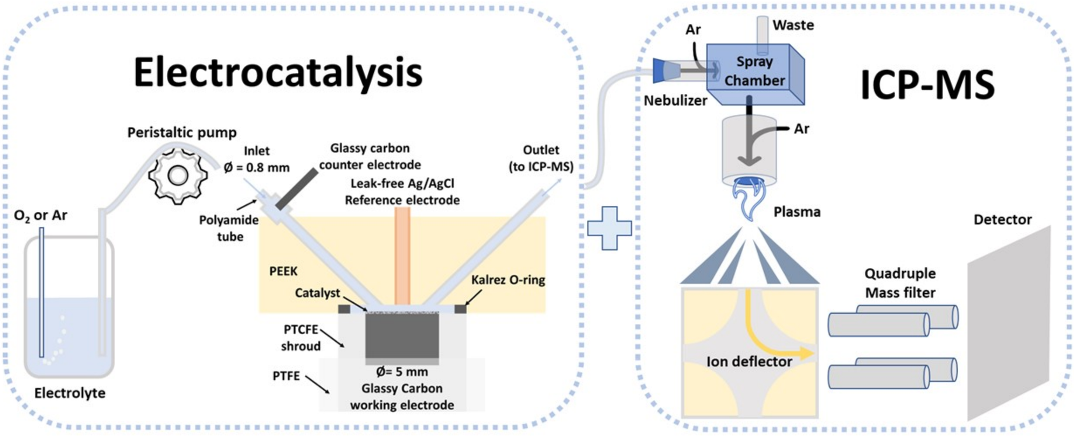

Figure S1. Schematic of flow cell setup with RDE disk electrode tip, Ag/AgCl/3.4 M Cl^- reference electrode and glassy carbon counter electrode coupled to online ICP-MS.

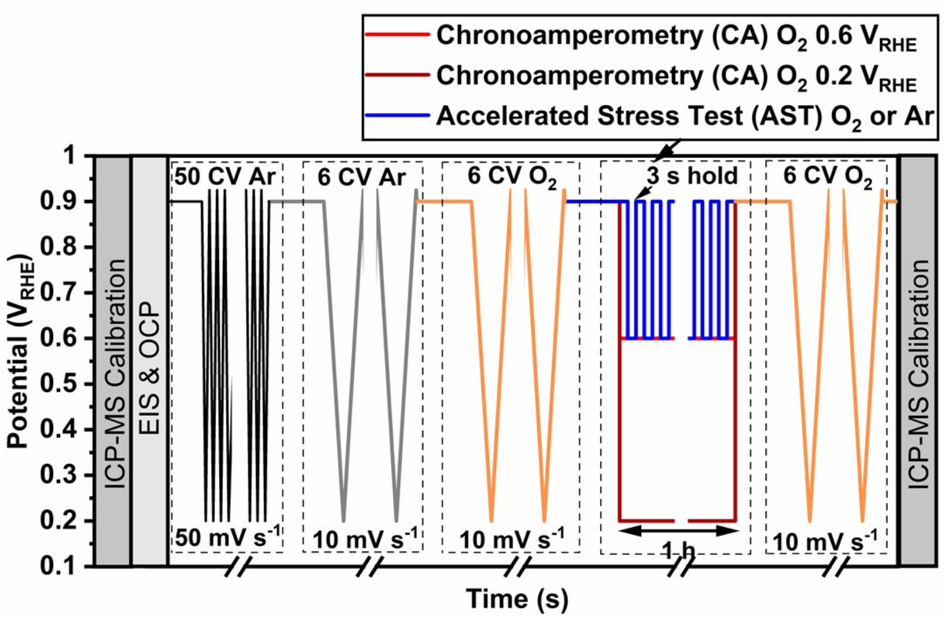

Figure S2. Protocol followed during flow cell electrochemical testing.

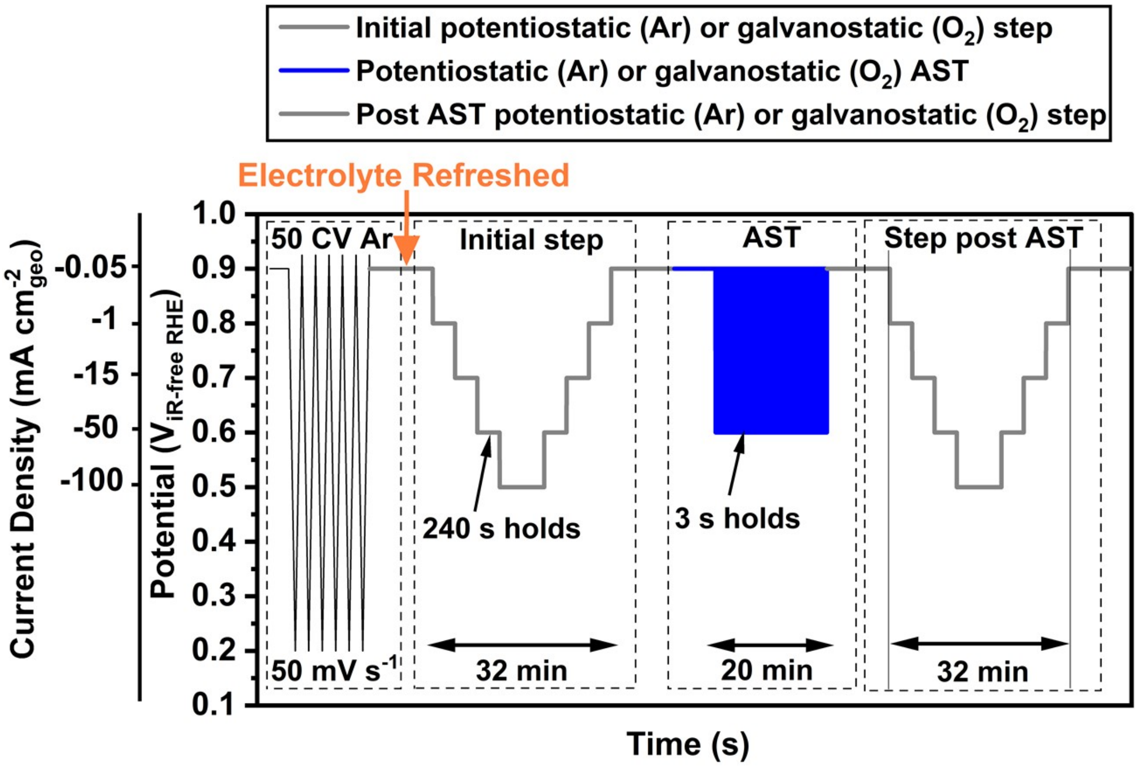

Figure S3. Protocol followed during GDE-ICP-MS testing. Note, the electrolyte in the GDE half-cell was refreshed between the 50 CV in Ar and the initial step and that the online measurements only started after the electrolyte was refreshed.

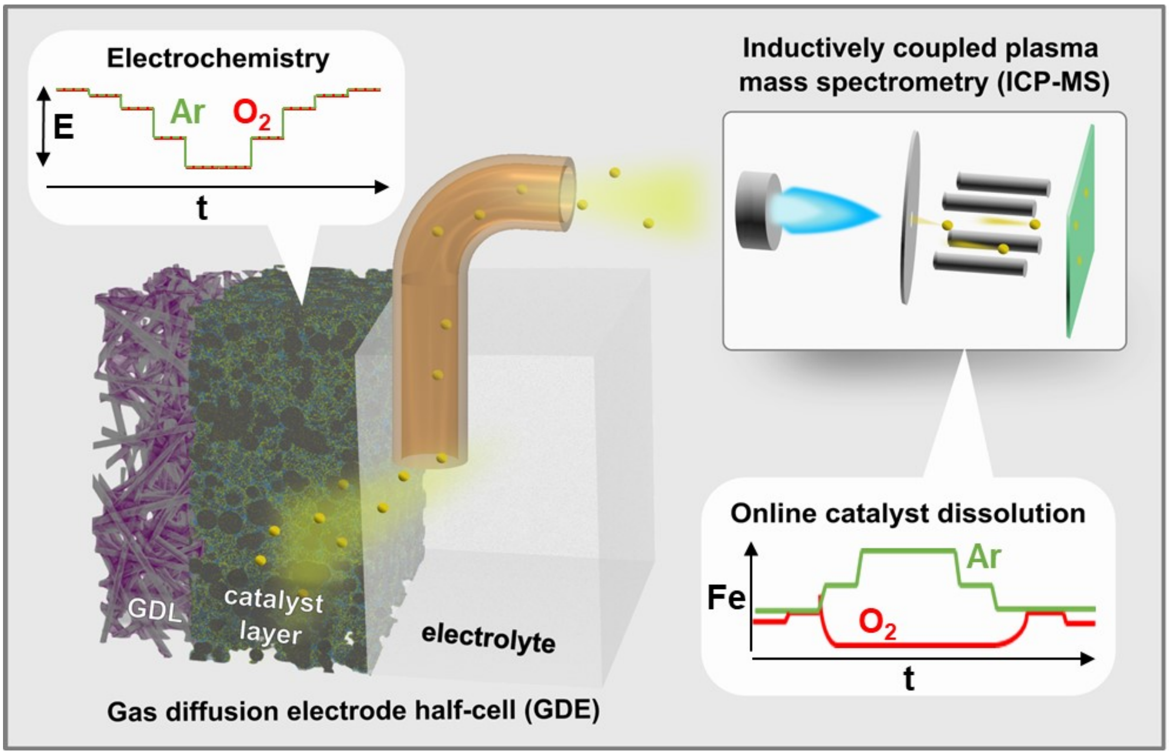

Figure S4. Schematic representing GDE coupled to ICP-MS. Adapted from Ehelebe *et al*. [13]

Table S1. Loading, flow rate and collection efficiency for GDE ICP-MS experiments.

Conditions	Loading (mg_{FeNC} $cm^{-2}{}_{geo}$)	Flow rate (mL min^{-1})	Collection efficiency (%)
Ar 20 ^{o}C	0.93	0.207	26.5
	0.93	0.207	35.5
O_2 20 ^{o}C	0.87	0.214	23.5
	1.03	0.203	28.7
O_2 75 ^{o}C	0.71	0.214	32.8
	0.82	0.226	29.4

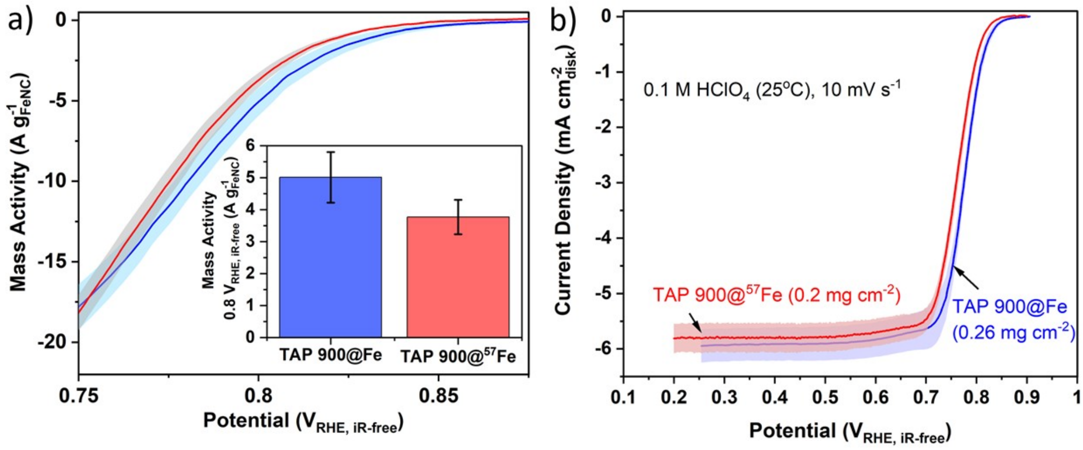

Figure S5. Comparison between TAP 900@Fe and TAP 900@^{57}Fe for cathodic scan in O_2-saturated 0.1 M HClO4. a.) Mass activity, with inset showing mass activity at 0.8 $V_{RHE,\ iR\text{-}free}$ b.) Current density. O_2 reduction in 0.1 M $HClO_4$ at 10 mV s^{-1} and 1600 rpm. O_2-Ar correction applied. TAP 900@Fe data re-plotted from previous work.[1] Error represents standard deviation from at least four repeat measurements, and is highlighted with red and blue colors.

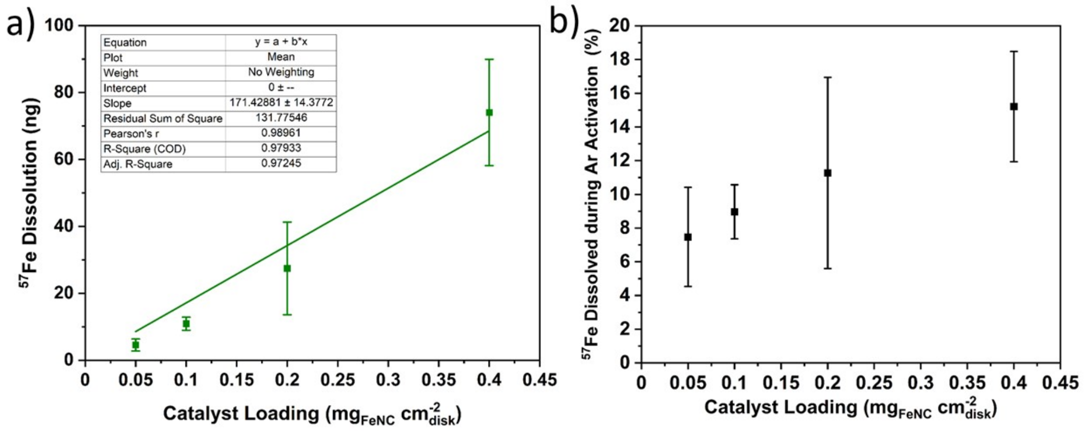

Figure S6. a.) Amount of ^{57}Fe dissolution with varying catalyst loading over cyclic voltammetry at 50 mV s^{-1}. Line of best fit is assumed to intercept the axis through the origin. b.) Corresponding percentage of total catalyst ^{57}Fe dissolved. Error bars represent standard deviation of at least four separate measurements.

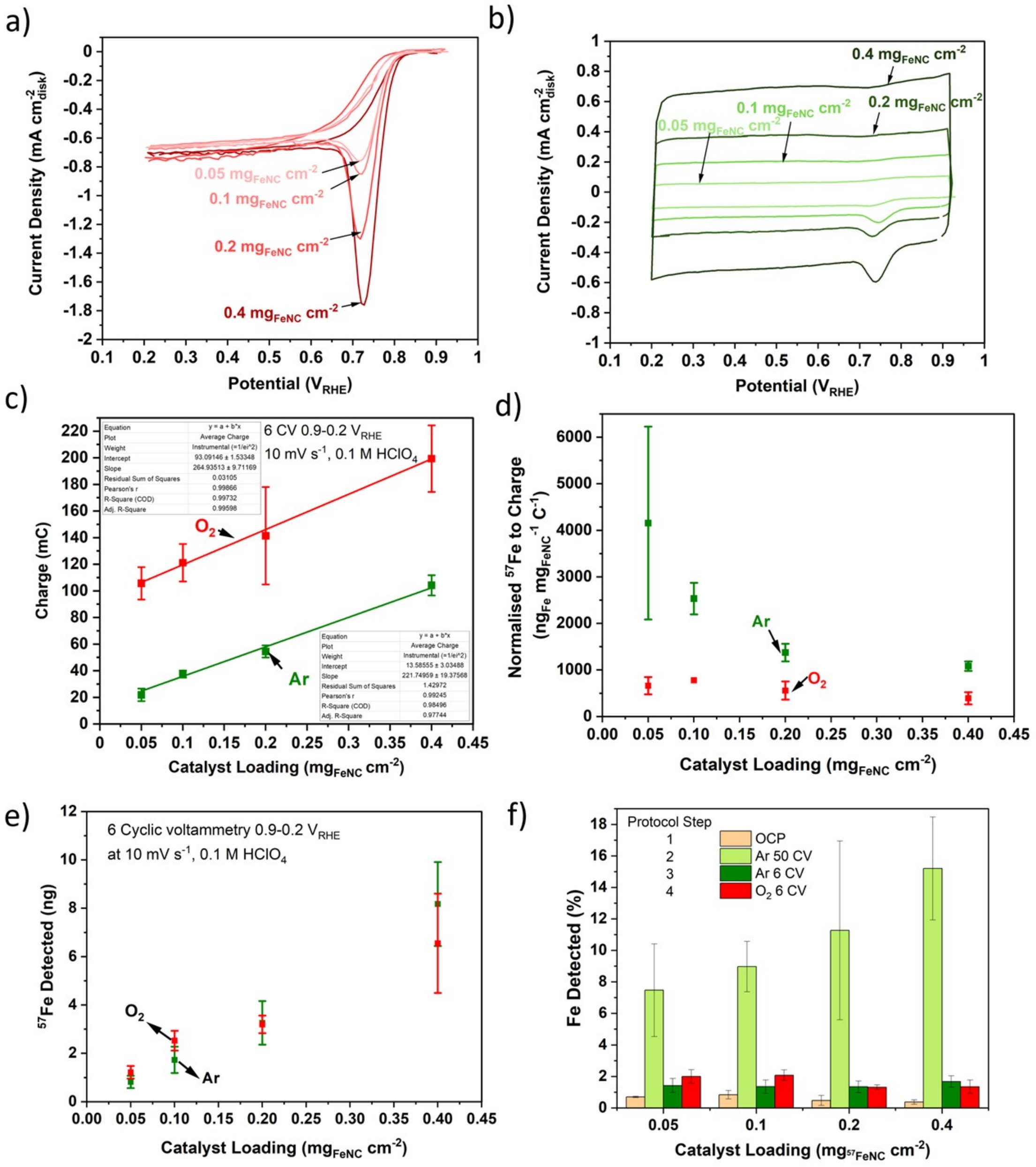

Figure S7. Flow cell under a.) O_2-saturated conditions, with O_2 - Ar correction applied. b.) Ar-saturated conditions. c.) Charge passed over six CV. d.) ^{57}Fe concentration normalized to charge passed and e.) ^{57}Fe mass at varying catalyst loading over six CV. f.) Percentage of ^{57}Fe detected at different stages of protocol. Error bars represent minimum of four repeat measurements.

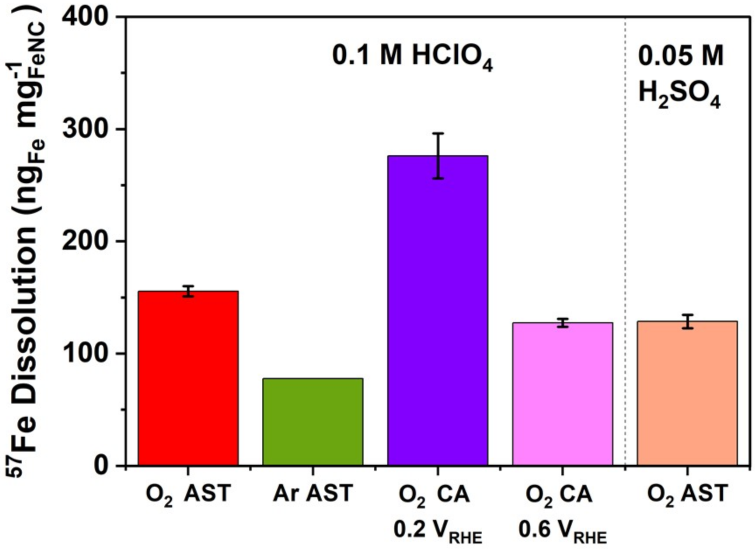

Figure S8. ^{57}Fe dissolution over different stability tests in flow cell. All tests over 1 h with 0.2 mg_{FeNC} cm^{-2}_{geo}.

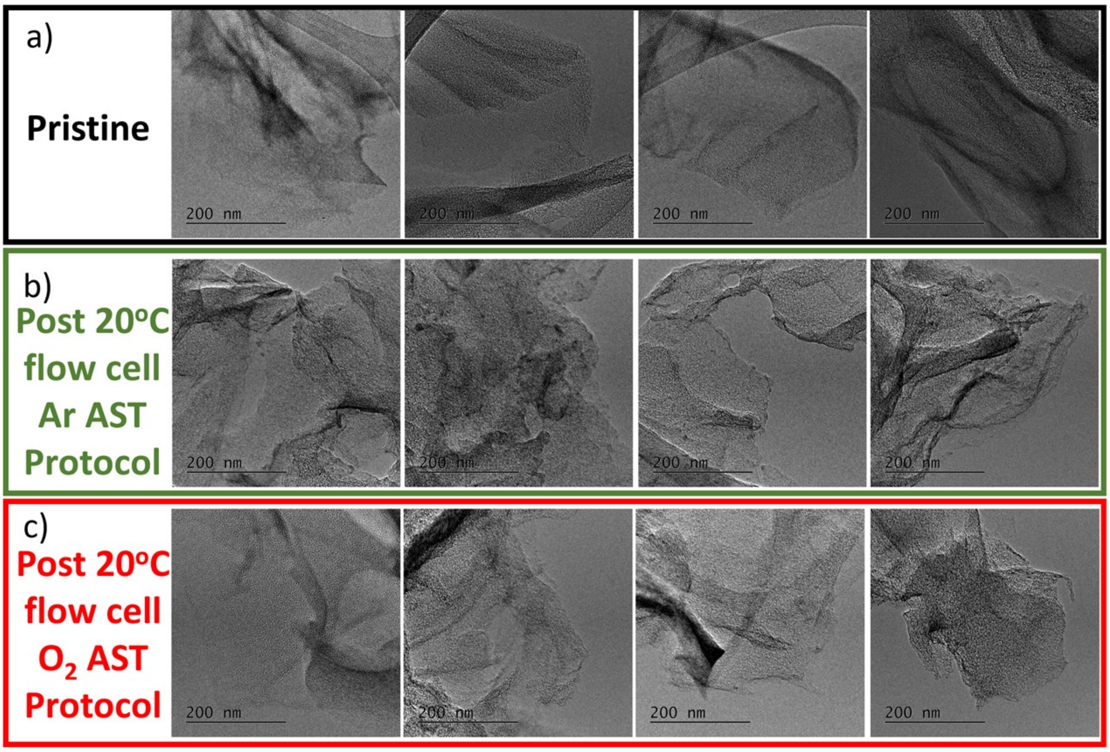

Figure S9. Bright-field TEM images of TAP 900@^{57}Fe a.) Pristine. After protocol with AST in flow cell with b.) Ar-saturation. c.) O_2-saturation.

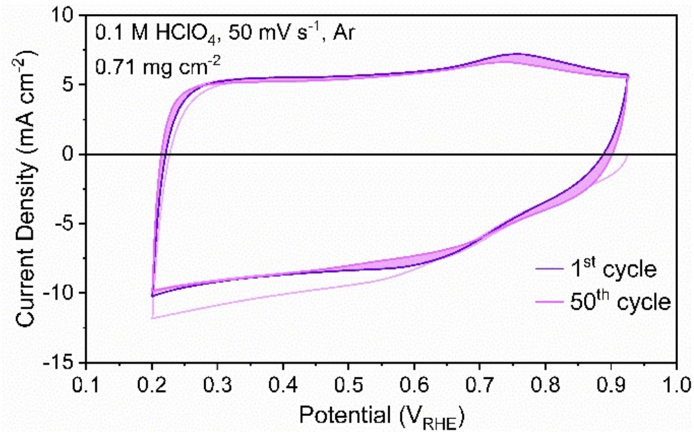

Figure S10. Example of the 50 CVs measured at 50 mV s^{-1} in Ar condition before online GDE-ICP-MS measurement. The Fe redox peak decreases with each cycle.

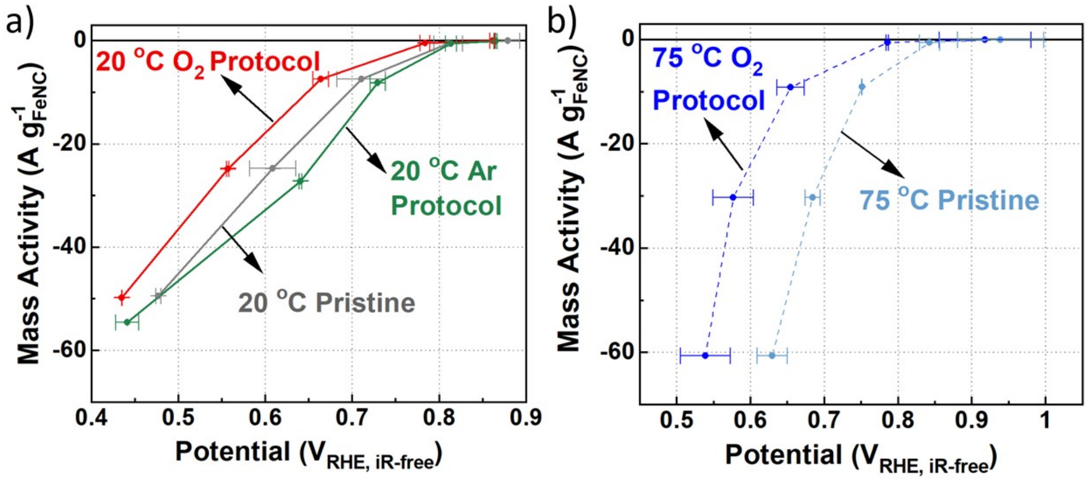

Figure S11. ORR mass activity of GDE at a.) 20 °C O_2 reduction in GDE for pristine TAP 900@Fe, and after O_2 and Ar 20 °C GDE protocols. b.) 75°C O_2 reduction in GDE for pristine TAP 900@Fe and after O_2 GDE 75 °C GDE protocol. Error represents two repeat measurements.

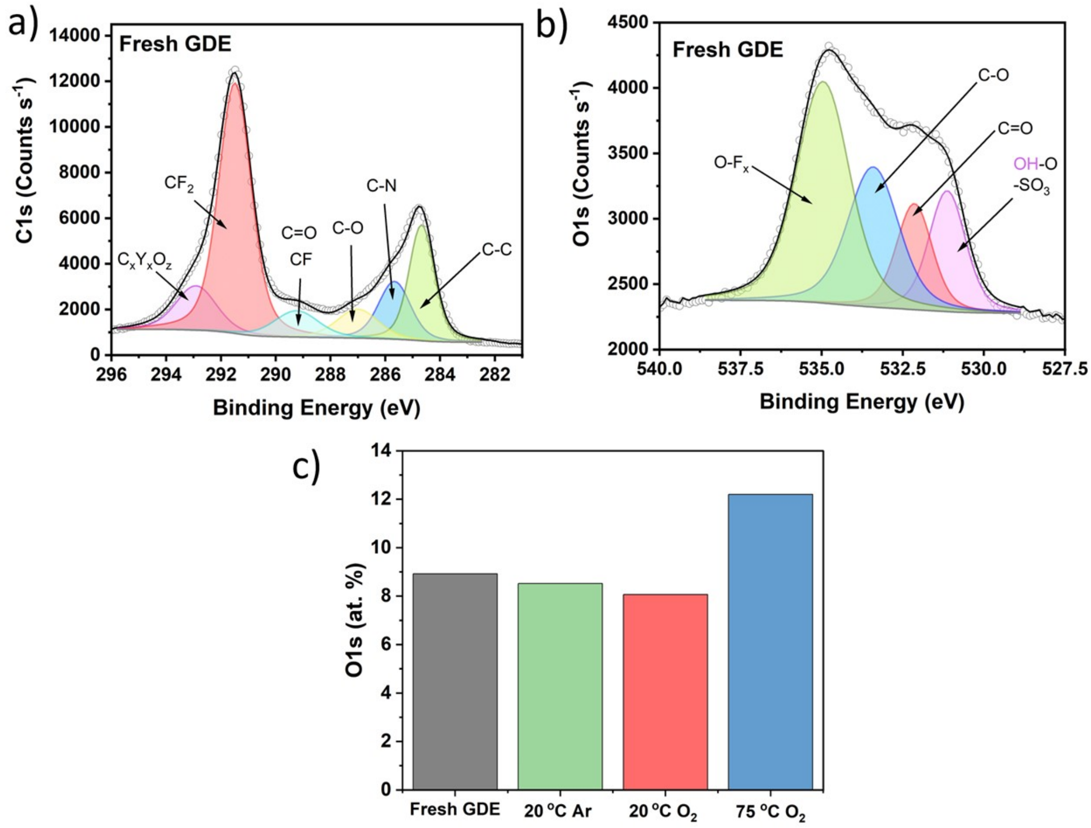

Figure S12. Peak fitting of fresh TAP 900@Fe GDE for a.) C1s and b.) O1s. c.) Total O1s content in at.% for GDE samples. The content is based on counts from only C and O.

Table S2. Relative content in at.% considering only C and O in GDE.

XPS Peak	Fresh	Post GDE 20 ^{o}C Ar	Post GDE 20 ^{o}C O_2	Post GDE 75 ^{o}C O_2
**C1s**	91.1	91.5	91.9	87.8
**O1s**	8.9	8.5	8.1	12.2

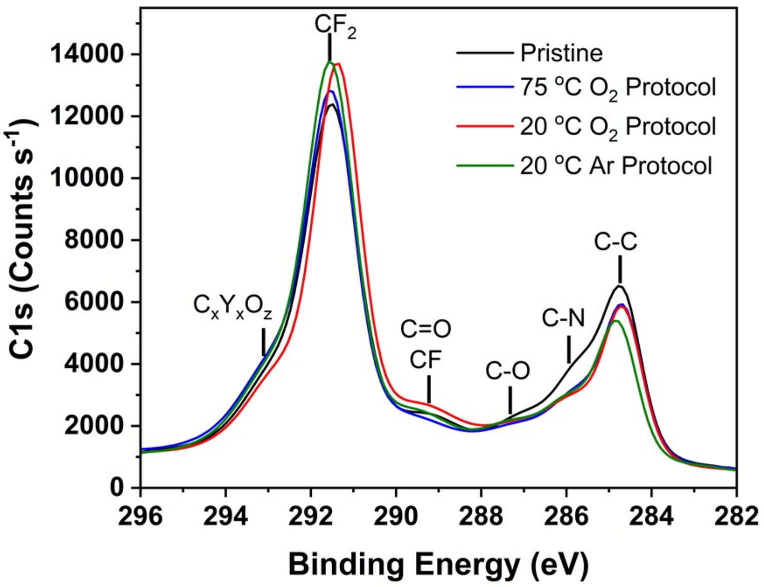

Figure S13. Comparison of X-ray photoelectron spectra (C1s band) for GDE samples.

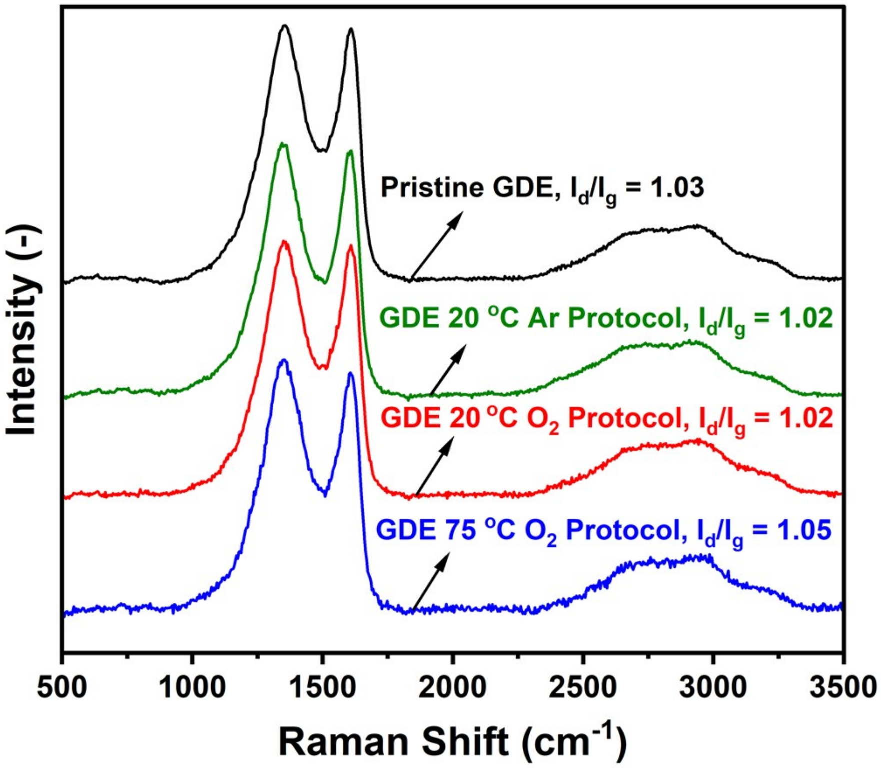

Figure S14. Raman spectra of pristine TAP 900@Fe on GDE, and post-GDE protocol at 20 ^oC (Ar and O_2) and 75 ^oC (O_2).

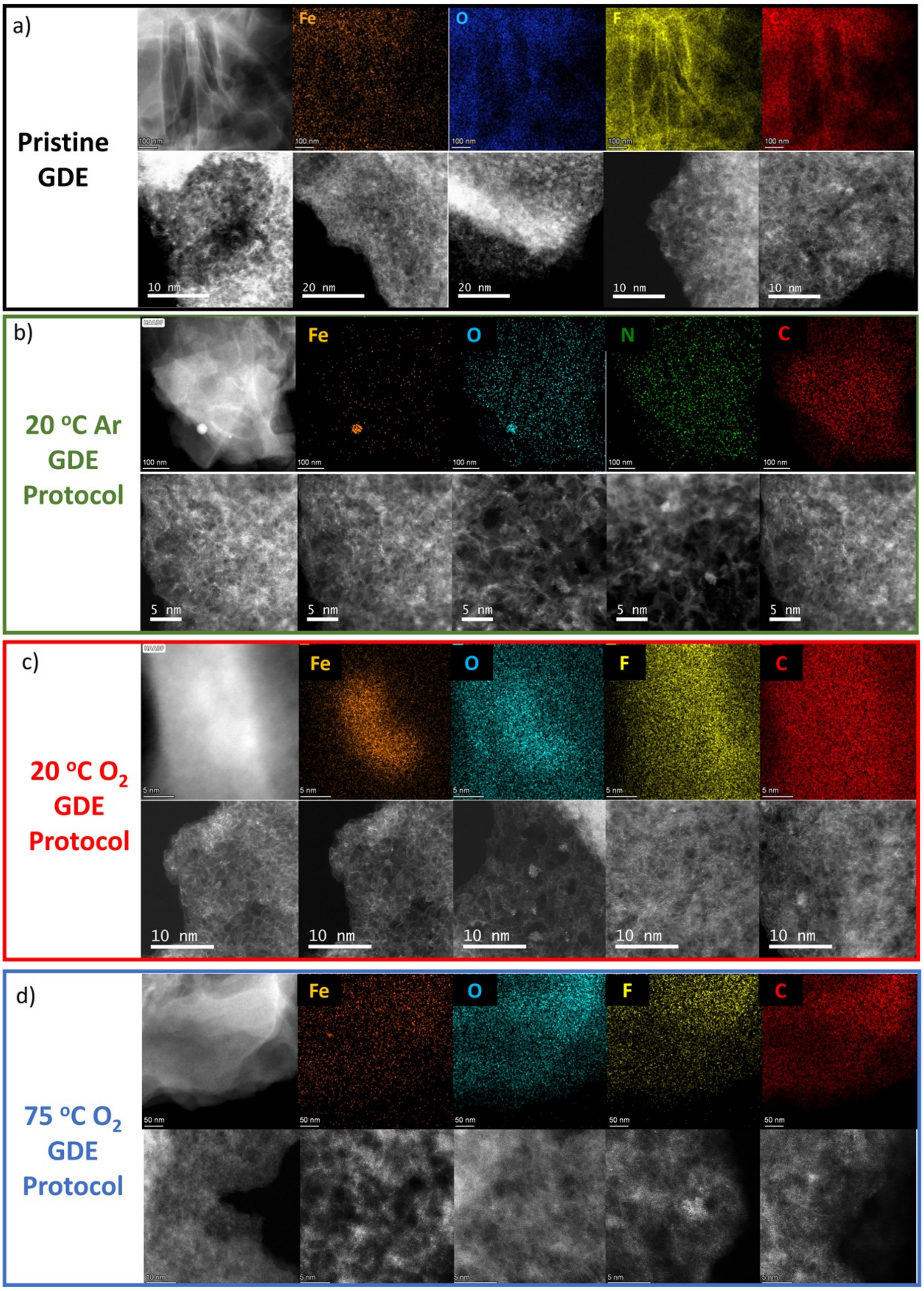

Figure S15. a.) STEM-EDX spectrum images (top) and HAADF-STEM (bottom) of a.) Pristine GDE b.) Post 20 °C Ar protocol c.) Post 20 °C O_2 protocol. d.) Post 75 °C O_2 protocol.

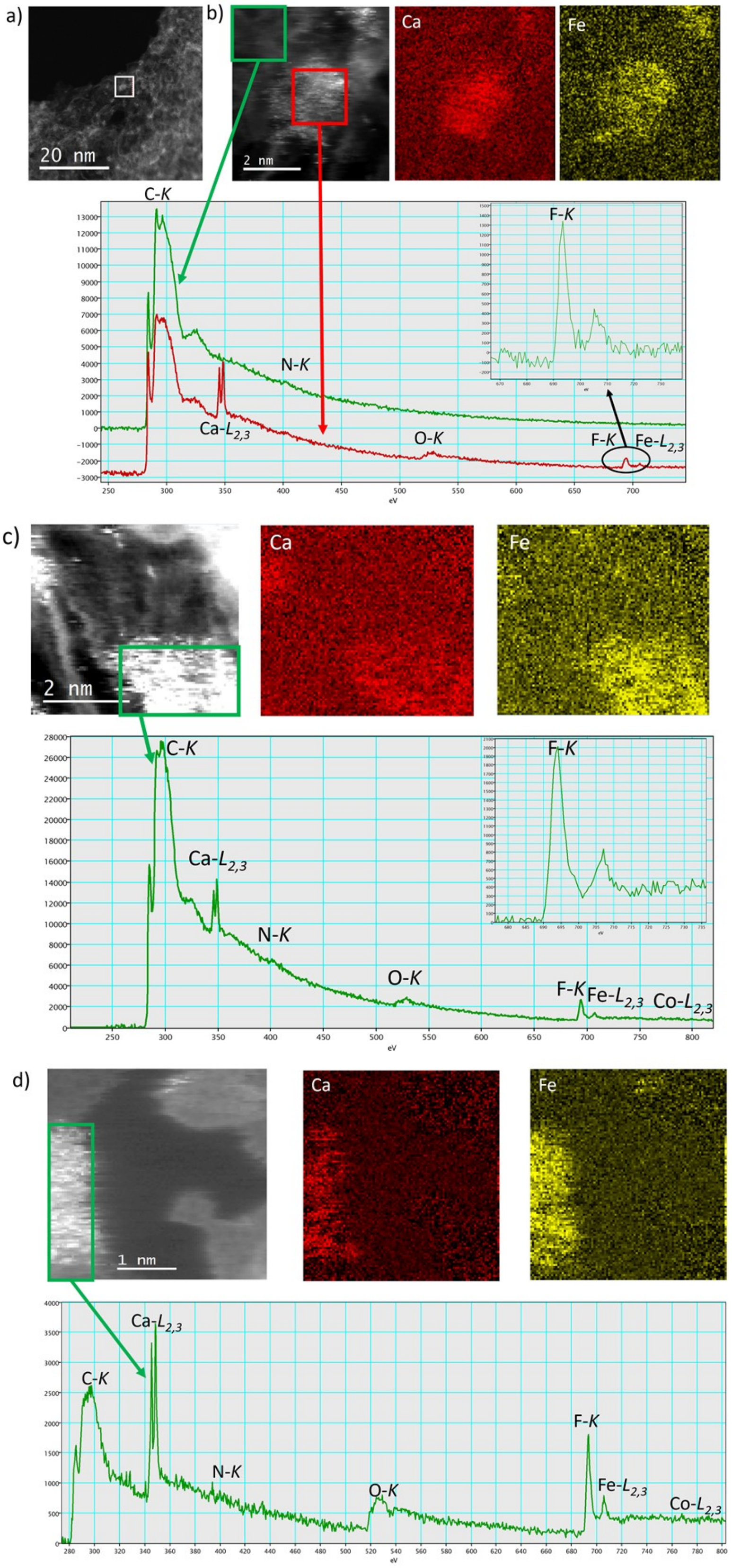

Figure S16. a.) HAADF-STEM of fresh TAP 900@Fe GDE b.) and HAADF-STEM of particle highlighted in white box in a.), with corresponding EDX spectrum image and EELS. HAADF-STEM and corresponding EDX spectrum image and EELS of GDE c.) Post 20 ^{o}C Ar protocol. d.) Post 20 ^{o}C O_2 protocol. The oxidation state of Fe and Ca may be influenced by beam damage so are not discussed.

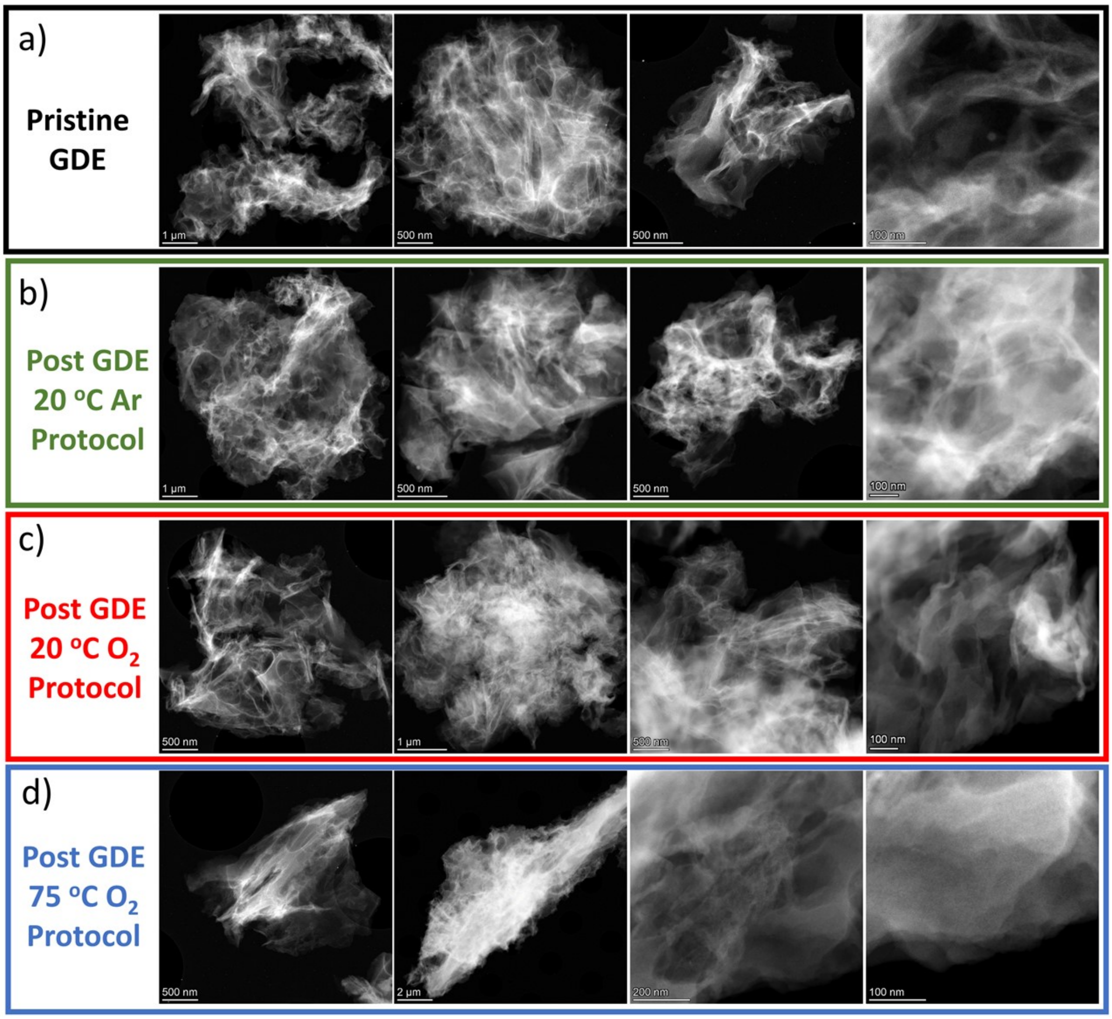

Figure S17. HAADF-STEM of TAP 900@Fe structure in a.) pristine GDE. Post GDE protocols in b.) 20 °C Ar. c.) 20 °C O_2. d.) 75 °C O_2.

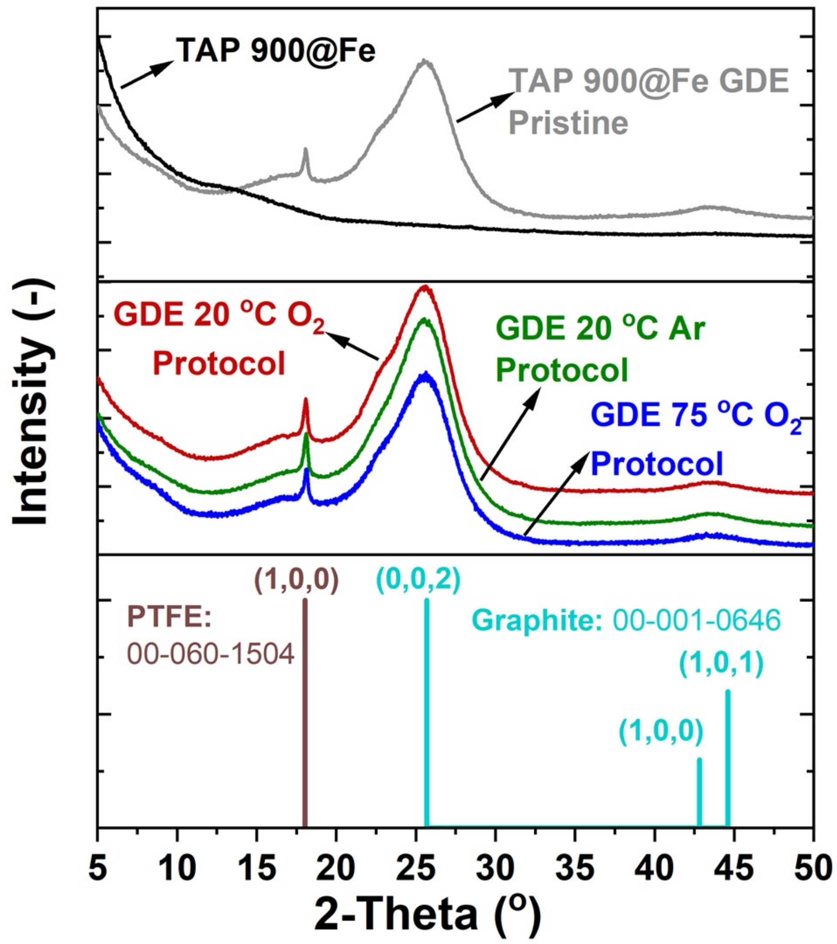

Figure S18. XRD of pristine TAP 900@Fe, prepared on GDE and post GDE protocols, with peaks of PTFE and graphite labelled.

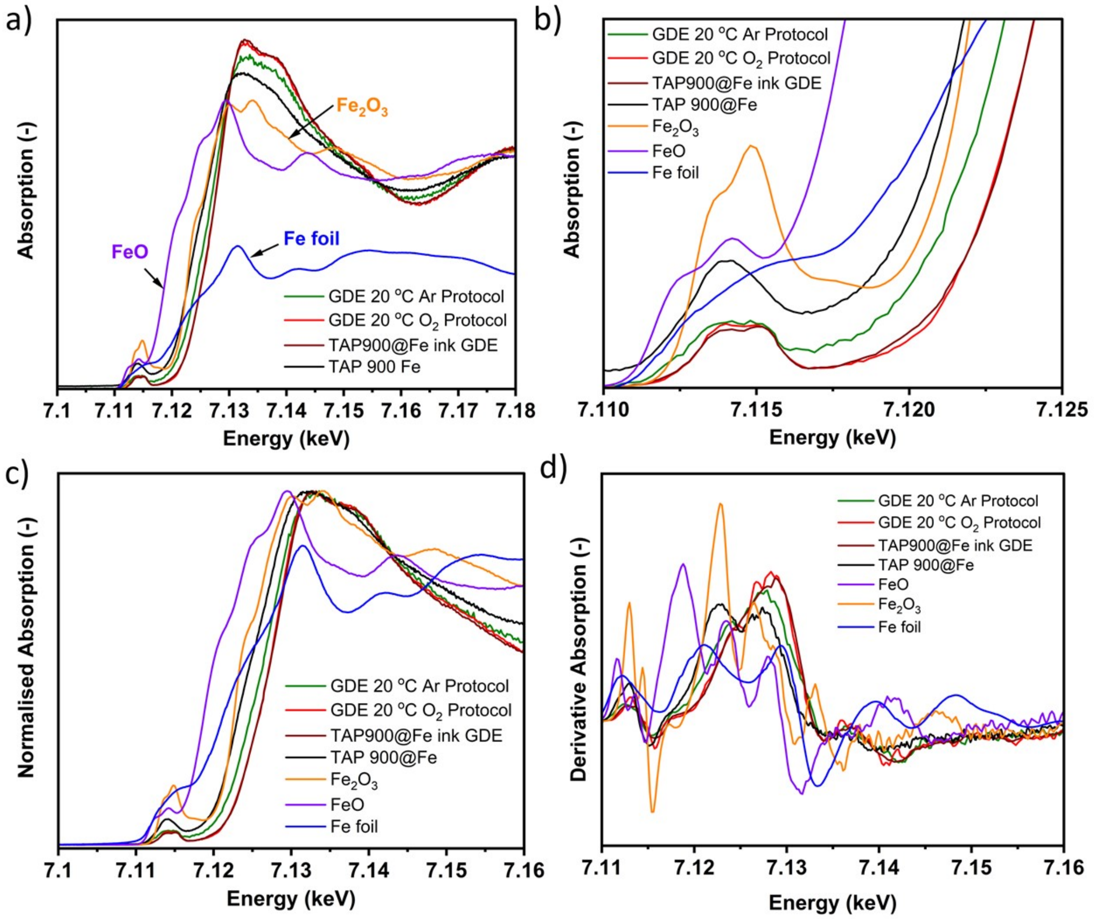

Figure S19. *Ex situ* XANES a.) Absorption and b.) pre-edge region c.) Normalised and d.) First derivative of TAP 900@Fe powder and GDE ink, post GDE 25°C Ar and O_2 protocols and reference FeO, Fe_2O_3 and Fe foil. Due to the difference in the experimental conditions of Fe foil and the other samples, the direct comparison of the absolute XANES intensities cannot be made for Fe foil. Instead, for Fe foil, focus is made on the shape of the XANES. To note in Figure a.) and b.) spectra are normalized by incoming beam and the area below the spectra, while in c.) an additional normalization is applied based on the maximum intensity.

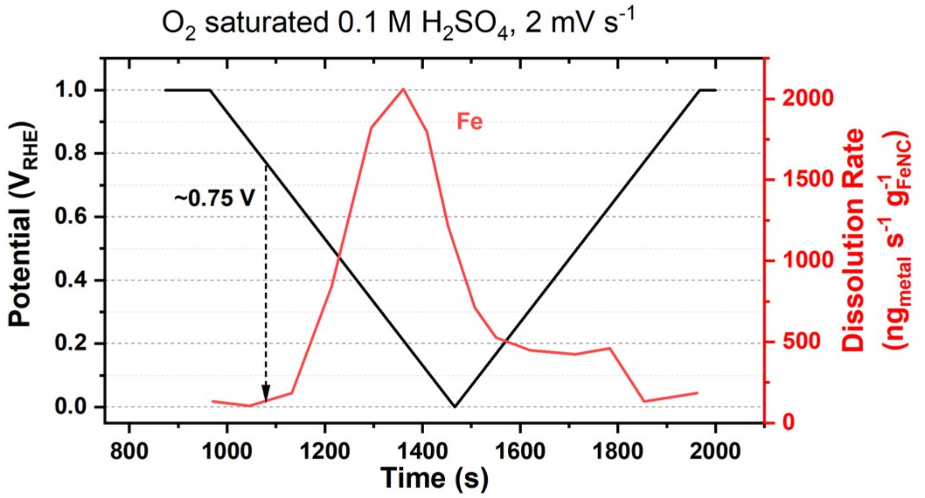

Figure S20. Online flow cell ICP-MS dissolution data replotted from Santori *et al*. [14] Ar-pyrolysed Fe-N-C under O_2-saturated 0.1 M H_2SO_4 at 2 mV s^{-1}.

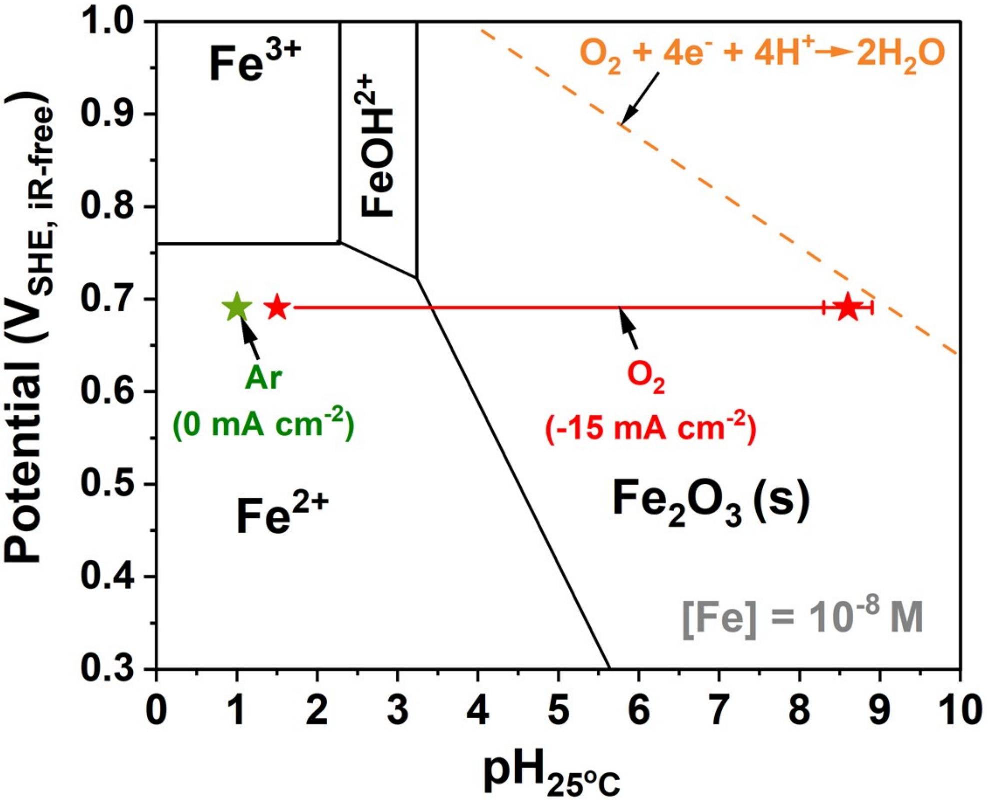

Figure S21. Pourbaix diagram of Fe at 25 ^{o}C and [Fe] = 10^{-8} M. Fe Pourbaix data replotted from Ref.[15] The error bar for O_2 represents variation of pH from different ε and k_r in Figure 5e.

References

1 J. Barrio, A. Pedersen, S. Ch. Sarma, A. Bagger, M. Gong, S. Favero, C. Zhao, R. Garcia-Serres, A. Y. Li, Q. Zhang, F. Jaouen, F. Maillard, A. Kucernak, I. E. L. Stephens and M. Titirici, *Adv. Mater.*, 2023, **35**, 2211022.

2 J. Barrio, A. Pedersen, J. Feng, S. Ch. Sarma, M. Wang, A. Y. Li, H. Yadegari, H. Luo, M. P. Ryan, M.-M. Titirici and Ifan. E. L. Stephens, *J. Mater. Chem. A*, 2022, **10**, 6023–6030.

3 A. Mehmood, J. Pampel, G. Ali, H. Y. Ha, F. Ruiz-Zepeda and T.-P. Fellinger, *Adv. Energy Mater.*, 2018, **8**, 1701771.

4 K. T. Santos, K. Kumar, L. Dubau, H. Ge, S. Berthon-Fabry, C. S. A. Vasconcellos, F. H. B. Lima, T. Asset, P. Atanassov, V. A. Saveleva, P. Glatzel, X. Li, F. Jaouen and F. Maillard, *J. Power Sources*, 2023, **564**, 232829.

5 H. Zhang, L. Osmieri, J. H. Park, H. T. Chung, D. A. Cullen, K. C. Neyerlin, D. J. Myers and P. Zelenay, *Nat. Catal.*, 2022, 571–592.

6 K. Artyushkova, M. J. Workman, I. Matanovic, M. J. Dzara, C. Ngo, S. Pylypenko, A. Serov and P. Atanassov, *ACS Appl. Energy Mater.*, 2018, **1**, 68–77.

7 D. K. Paul, J. B. Giorgi and K. Karan, *J. Electrochem. Soc.*, 2013, **160**, F464.

8 Y.-P. Ku, K. Ehelebe, A. Hutzler, M. Bierling, T. Böhm, A. Zitolo, M. Vorokhta, N. Bibent, F. D. Speck, D. Seeberger, I. Khalakhan, K. J. J. Mayrhofer, S. Thiele, F. Jaouen and S. Cherevko, *J. Am. Chem. Soc.*, 2022, **144**, 9753–9763.

9 M. Rouhet, S. Bozdech, A. Bonnefont and E. R. Savinova, *Electrochem. Commun.*, 2013, **33**, 111–114.

10 P. Vanýsek, *CRC Handbook of Chemistry and Physics*, CRC Press, Boca Raton, USA, 92nd edn., 2011.

11 J. Newman and K. E. Thomas-Alyea, *Electrochemical Systems*, John Wiley & Sons, Third., 2004.

12 B. Tjaden, S. J. Cooper, D. J. Brett, D. Kramer and P. R. Shearing, *Curr. Opin. Chem. Eng.*, 2016, **12**, 44–51.

13 K. Ehelebe, J. Knöppel, M. Bierling, B. Mayerhöfer, T. Böhm, N. Kulyk, S. Thiele, K. J. J. Mayrhofer and S. Cherevko, *Angew. Chem. - Int. Ed.*, 2021, **60**, 8882–8888.

14 P. G. Santori, F. D. Speck, J. Li, A. Zitolo, Q. Jia, S. Mukerjee, S. Cherevko and F. Jaouen, *J. Electrochem. Soc.*, 2019, **166**, F3311–F3320.

15 B. Beverskog and I. Puigdomenech, *Corros. Sci.*, 1996, **38**, 2121–2135.
